# Oncogenic role of MiR-130a in oral squamous cell carcinoma

**DOI:** 10.1038/s41598-021-87388-4

**Published:** 2021-04-08

**Authors:** Karthik Mallela, Swamy Shivananda, Kodaganur S. Gopinath, Arun Kumar

**Affiliations:** 1 Department of Molecular Reproduction, Development and Genetics, Indian Institute of Science, Bangalore, 560012 India; 2 HCG-Bangalore Institute of Oncology, Bangalore, 560027 India

**Keywords:** Cancer, Oncology

## Abstract

Aberrant activation of the PI3K/AKT/mTOR pathway is attributed to the pathogenesis of oral squamous cell carcinoma (OSCC). In recent years, increasing evidence suggests the involvement of microRNAs (miRNAs) in oral carcinogenesis by acting as tumor suppressors or oncogenes. TSC1, as a component of the above pathway, regulates several cellular functions such as cell proliferation, apoptosis, migration and invasion. Downregulation of TSC1 is reported in oral as well as several other cancers and is associated with an unfavourable clinical outcome in patients. Here we show that oncogenic miR-130a binds to the 3′UTR of TSC1 and represses its expression. MiR-130a-mediated repression of TSC1 increases cell proliferation, anchorage independent growth and invasion of OSCC cells, which is dependent on the presence of the 3′UTR in TSC1. We observe an inverse correlation between the expression levels of miR-130a and TSC1 in OSCC samples, suggesting that their interaction is physiologically relevant. Delivery of antagomiR-130a to OSCC cells results in a significant decrease in xenograft size. Taken together, the findings of the study indicate that miR-130a-mediated TSC1 downregulation is not only a novel mechanism in OSCC, but also the restoration of TSC1 levels by antagomiR-130a may be a potential therapeutic strategy for the treatment of OSCC.

## Introduction

Oral squamous cell carcinoma (OSCC) is the most common malignancy of the head and neck (excluding non-melanoma skin cancer), with an estimated 354,864 new cases and 177,384 deaths reported annually (GLOBOCAN, 2018; https://gco.iarc.fr/today/home). India accounts for 34% of the world oral cancer burden with an age-standardized incidence rate (ASR) of 13.9 and 4.3 per 100,000 males and females respectively (GLOBOCAN, 2018). OSCC is the leading malignancy in males and the 4^th^ most common cancer in females, and accounts upto 10.4% of all cancers in India. Despite advancements in the treatment strategies, there is hardly any improvement in the 5-year survival rate of OSCC patients which ranges from 34% to 62.9%, thereby necessitating the identification of new molecular markers and signaling pathways for better prognosis and therapeutic intervention of OSCC^1^.

Dysregulation of PI3K/AKT/mTOR signaling axis, which regulates many facets of cellular functioning including cell proliferation, survival, apoptosis, metabolism and angiogenesis, is associated with the pathogenesis of OSCC^2,3^. The TSC1-TSC2-TBC1D7 complex negatively regulates this axis by inhibiting the activation of mTORC1 through a small GTPase protein RHEB^4,5^. Mutations in the tumor suppressor gene *TSC1* cause an autosomal dominant disorder, Tuberous sclerosis complex (TSC), characterized by intellectual disability and benign tumor formations in various organs including kidneys, brain, heart, retina and skin^6^.

The *TSC1* gene, located on chromosome 9q34.13, codes for a 130 kDa protein hamartin, which is ubiquitously expressed in all the tissues^6^. TSC1 plays a crucial role in facilitating multiple cellular functions including cell proliferation, adhesion and migration, autophagy, angiogenesis and tumor development^7–12^. Interestingly, TSC1 is also involved in centrosome maturation and/or mitotic progression by interacting with CDC2/cyclinB1 and PLK1^13,14^. It links Insulin-AKT signaling to the TGF-β-Smad2/3 pathway where it is not only required for TGF-β1-induced growth arrest but also for TGF-β1-induced epithelial-mesenchymal transition (EMT)^15^.

Several deletion and knockout studies of TSC1 have established its tumor suppressive role in various cancers. For example, *TSC1* homozygous deletion mutant mice (*Tsc1-/-*) are embryonic lethal, while heterozygous *TSC1* mutant mice (*Tsc1* ±*)* develop renal cell carcinomas and benign tumors in several organs^16^. Kladney et al. showed that conditional inactivation of *TSC1* is sufficient to activate the prostatic neoplasia-associated signaling cascade and facilitate malignant transformation^17^. A recent study using a constitutive *TSC1* transgenic (Tsc1^tg^) mouse model showed that moderate overexpression of TSC1 enhances overall health, particularly cardiovascular health and thereby improves survival^18^. Furthermore, TSC1 is downregulated in several cancers, such as cancers of the breast, prostate, oral cavity, bladder, liver and lung^2,17,19–23^. More importantly, a low expression of TSC1 is associated with an unfavorable clinical outcome in patients with breast, gastric and colorectal tumors^19,24,25^. Collectively, the above observations implicate that TSC1 has a potential role in tumorigenesis and suggest that it could be an attractive target for anti-cancer therapy.

Accumulating evidence suggests the emerging role of microRNAs (miRNAs) in the pathogenesis of OSCC by acting as oncogenes or tumor suppressors^26^. MiRNAs, as post-transcriptional gene regulators, affect a myriad of cellular processes like proliferation, apoptosis, invasion, angiogenesis and autophagy, thus playing a significant role in different stages of tumor development and progression, including OSCC^26^. Interestingly, many reports show that each tumor type has a distinct miRNA signature that distinguishes it from other cancers and normal tissues which can be exploited. Therefore, miRNAs offer an attractive potential strategy for early cancer detection, prognosis and therapeutics^27^. Since the downregulation of *TSC1* is reported in several malignancies, including OSCC, the current study aimed to explore its regulation through miRNAs^2,19–23^. Here we report that an oncogenic miR-130a regulates the expression of tumor suppressor gene *TSC1*. We further show that a synthetic antagomiR-130a may be used to restore the level of TSC1, which could be used as an effective strategy to treat OSCC.

## Results

### Regulation of TSC1 by miR-130a

**Figure 1:**
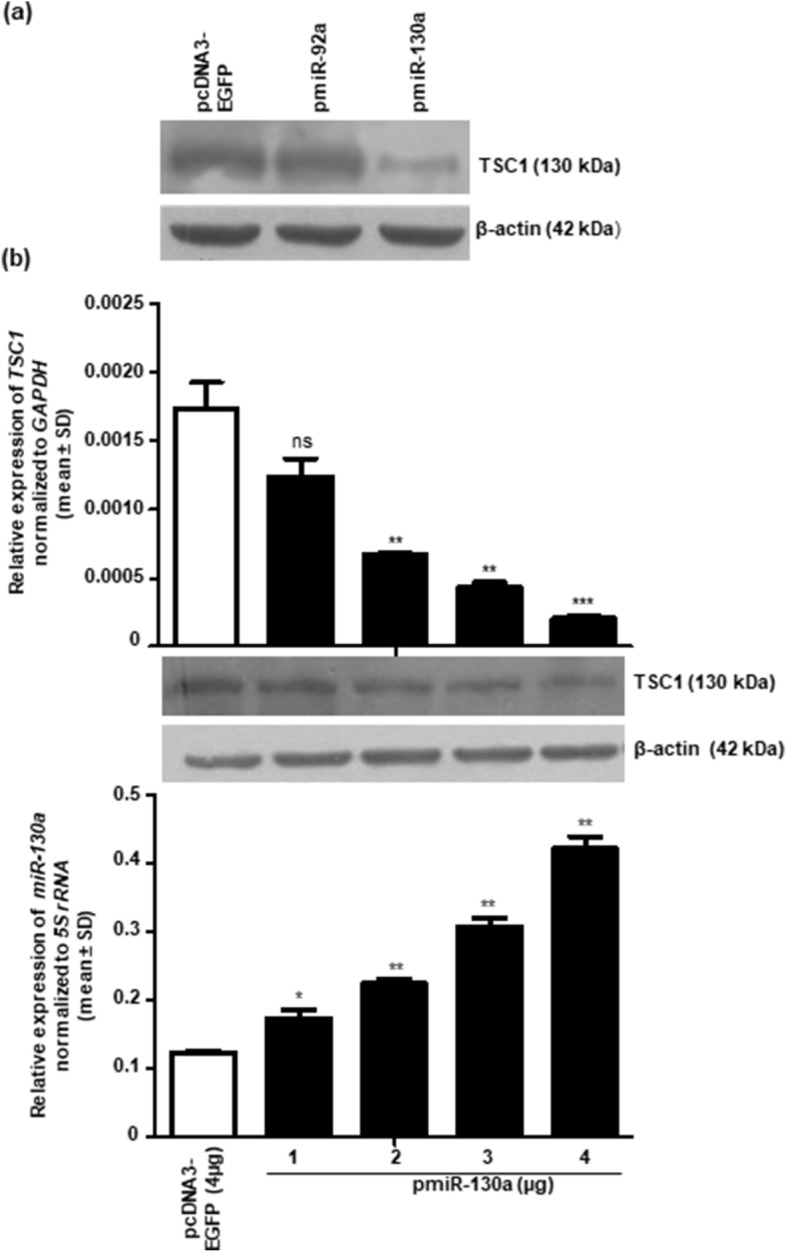
Regulation of TSC1 by miR-130a. (**a**) The Western blot analysis shows the effect of overexpression of miR-92a and miR-130a on the level of TSC1 in SCC084 cells. Cells were transfected with 4 μg of empty vector pcDNA3-EGFP or constructs harboring different miRNAs cloned in this vector. Note, the reduced level of TSC1 in cells transfected with pmiR-130a (n = 2). (**b**) Dose-dependent reduction in the level of TSC1 at transcript and protein levels, following overexpression of miR-130a in SCC084 cells (n = 2). RT-qPCR data for *TSC1* and *miR-130a* expression are an average of 2 technical replicates. *, *p* < 0.05; **, *p* < 0.01; ***, *p* < 0.001; and ns, non-significant (full-length blots are presented in Supplementary Figure S5).

**Figure 2:**
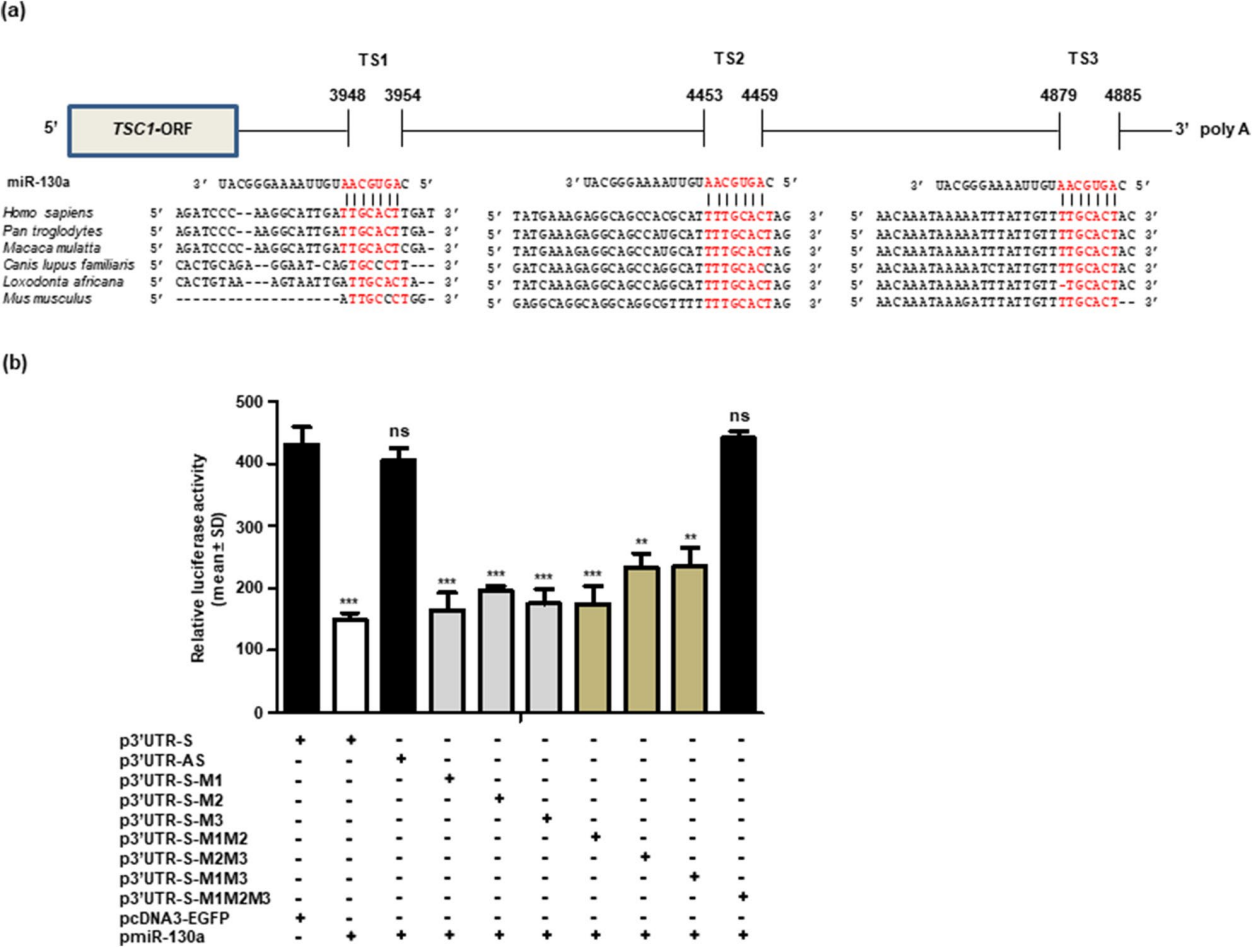
Confirmation of miR-130a binding to TSC1 3′UTR. (**a**) ClustalW alignment to show the conservation of putative miR-130a target sites (TSs) in the 3′UTR of TSC1 across different vertebrate species. (**b**) Confirmation of miR-130a binding to the TSC1 3′UTR by the dual luciferase reporter assay. Note, a significantly reduced luciferase activity in cells co-transfected with pmiR-130a and p3′UTR-S in comparison with those co-transfected with p3′UTR-S and pcDNA3- EGFP, confirming the binding of miR-130a to the 3′UTR of TSC1. Each bar for luciferase activity is the average of 3 biological replicates. **, p < 0.01; ***, p < 0.001; and ns, non-significant.

Five different miRNA target prediction programs (viz., DIANA-microT v3.0, microRNA, miRDB, TargetScan and PicTar) were used to identify the miRNAs that may target *TSC1.* We found several miRNAs with potential binding sites in the 3′UTR of *TSC1* (Supplementary Table S1) and preferentially picked up miR-130a and miR-92a for validation because most of the target prediction algorithms predicted them to target *TSC1* (Supplementary Table S1). To confirm if these miRNAs regulate the expression of *TSC1*, we cloned these miRNAs in the pcDNA3-EGFP vector and overexpressed in SCC084 cells. A significant decrease in TSC1 levels were observed in miR-130a transfected cells but not in miR-92a transfected cells (Fig. 1a). Furthermore, miR-130a downregulated the level of *TSC1* in SCC084 cells in a dose-dependent manner at both the transcript and protein levels (Fig. 1b). This suggested that miR-130a post-transcriptionally represses the expression of *TSC1.* The bioinformatics analysis predicted three putative target sites (TS1, TS2 and TS3) for the miR-130a seed region within the 3′UTR of *TSC1*. The ClustalW alignment of target sites (TSs) for miR-130a in the 3′UTR of *TSC1* showed their conservation across different species (Fig. 2a), suggesting that they may have a prominent role to play in the regulation of *TSC1* expression.

To confirm the interaction between miR-130a and the 3′UTR of *TSC1*, we co-transfected pmiR-130a with p3′UTR-S (3′UTR of *TSC1* in a sense orientation) or p3′UTR-AS (3′UTR of *TSC1* in an antisense orientation, and thus lacking the miR-130a TSs), and p3′UTR-S and the vector pcDNA3-EGFP in SCC084 cells and quantitated the luciferase activity. A significant decrease in luciferase reporter activity was found in cells co-transfected with pmiR-130a and p3′UTR-S as compared with those co-transfected with p3′UTR-S and pcDNA3-EGFP (Fig. 2b) or pmiR-130a and p3′UTR-AS (Fig. 2b), confirming that miR-130a binds to the 3′UTR of *TSC1*. To determine whether all the three TSs in the 3′UTR of *TSC1* are functional for the binding of miR-130a, we mutated the three TSs individually or in combinations by site-directed mutagenesis. We then co-transfected the p3′UTR-S-M1 (mutated TS1, wild-type TS2 & TS3), p3′UTR-S-M2 (mutated TS2, wild-type TS1 & TS3), p3′UTR-S-M3 (mutated TS3, wild-type TS1 & TS2), p3′UTR-S-M1M3 (mutated TS1 & TS3, wild-type TS2), p3′UTR-S-M1M2 (mutated TS1 & TS2, wild-type TS3), p3′UTR-S-M2M3 (mutated TS2 & TS3, wild-type TS1) and p3′UTR-S-M1M2M3 (all three mutated TS1, TS2 & TS3) separately with pmiR-130a in SCC084 cells and quantitated the luciferase activity. The co-transfection of single mutant constructs (p3′UTR-S-M1, p3′UTR-S-M2 & p3′UTR-S-M3) or double mutant constructs (p3′UTR-S-M1M2, p3′UTR-S-M1M3 & p3′UTR-S-M2M3) with pmiR-130a showed a significant decrease in the luciferase activity compared with those co-transfected with p3′UTR-S and pcDNA3-EGFP (Fig. 2b)*.* No significant difference in luciferase activity was found between cells co-transfected with p3′UTR-S-M1M2M3 and pmiR-130a compared with those co-transfected with p3′UTR-S and pcDNA3-EGFP (Fig. 2b). All these observations clearly show that miR-130a regulates the expression of *TSC1* by interacting with all the three TSs in a site-specific manner.

### Effect of miR-130a-mediated regulation of *TSC1* on the PI3K/AKT/mTOR pathway

**Figure 3:**
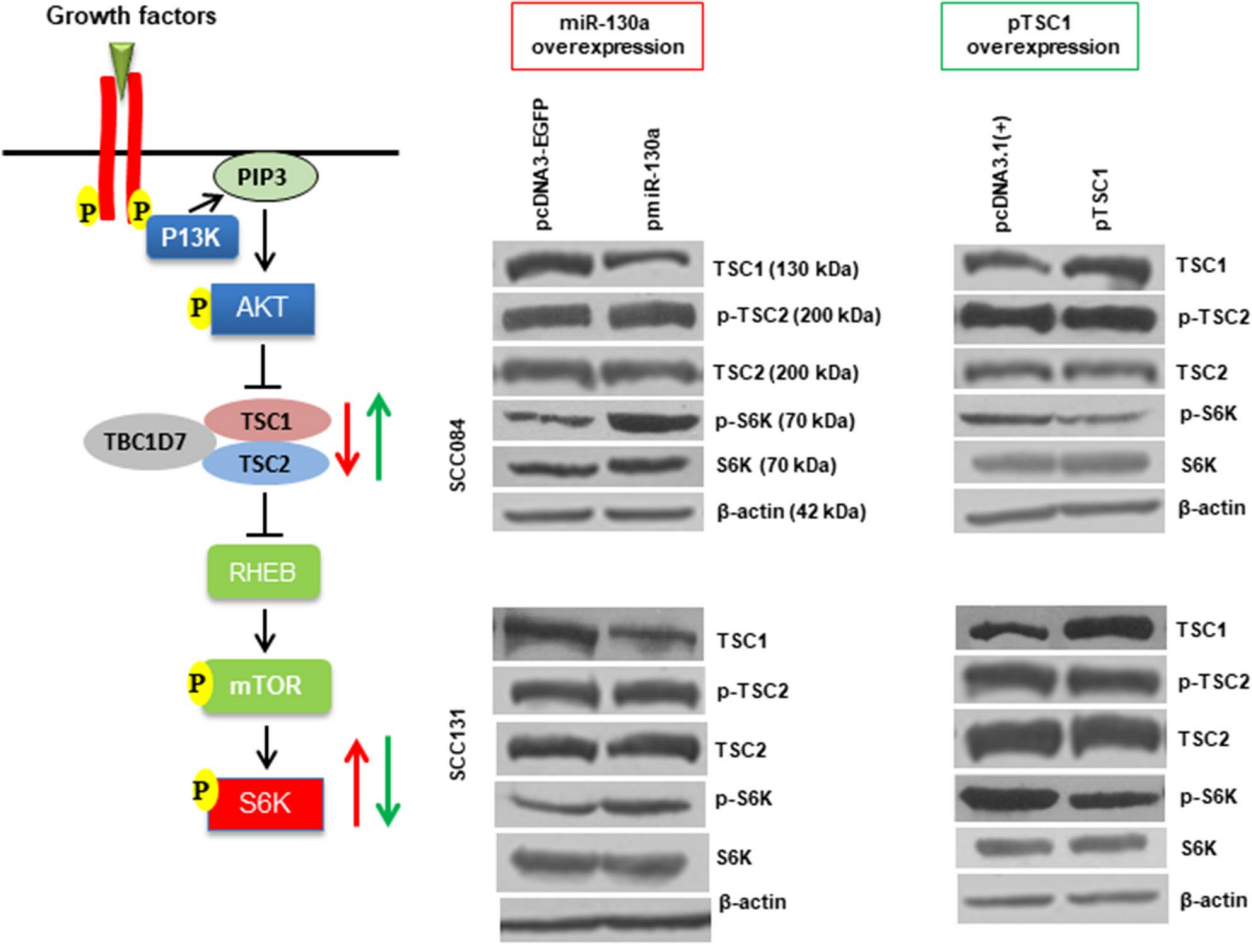
Effect of miR-130a-mediated regulation of TSC1 on the PI3K/AKT/mTOR pathway. Left panel: Diagrammatic representation of the PI3K/AKT/mTOR pathway. This figure was drawn using Microsoft PowerPoint version 2010. This figure is analysis adapted from Jin et al.^51^ and Jung et al.^52^. Middle panel: The Western blot analysis showed low TSC1 and high p-S6K levels in miR-130a transfected SCC084 and SCC131 cells as compared to vector transfected cells (n = 2). Right panel: The Western blot showed increased TSC1 and lower p-S6K levels in pTSC1 transfected cells as compared to vector transfected cells (n = 2) (full-length blots are presented in Supplementary Figure S6).

TSC1 as a vital component of the PI3K/AKT/mTOR pathway negatively regulates mTORC1 activity and plays an important role in many cellular processes like cell growth, proliferation and survival. We therefore wanted to test whether the overexpression of miR-130a or TSC1 in OSCC cells has a functional relevance in cell proliferation as a result of reduced or increased levels of TSC1, respectively. Hence, we transfected both SCC084 and SCC131 cells with pmiR-130a or pTSC1 constructs and analyzed the expression of several components of the PI3K/AKT/mTOR pathway. We observed an increased level of phospho-S6K (p-S6K) (a measure of mTORC1 activity) in SCC084 and SCC131 cells transfected with miR130a, and a lower level of p-S6K in cells transfected with pTSC1 compared to cells transfected with the vector only (Fig. 3). However, there was no change in the level of p-TSC2, total TSC2 and total S6K in miR-130a or pTSC1 transfected SCC084 and SCC31 cells compared to the vector control (Fig. 3). Also, the cell proliferation as measured by the BrdU cell proliferation assay was significantly higher in miR-130a transfected cells (Supplementary Fig. S1a) as compared to vector transfected cells, whereas pTSC1 transfected cells showed significantly reduced cell proliferation as compared to vector transfected cells (Supplementary Fig. S1b). These observations suggest that miR-130a-mediated regulation of *TSC1* activates the PI3K/AKT/mTOR pathway.

### MiR-130a is upregulated in OSCC samples with downregulation of *TSC1*

**Figure 4:**
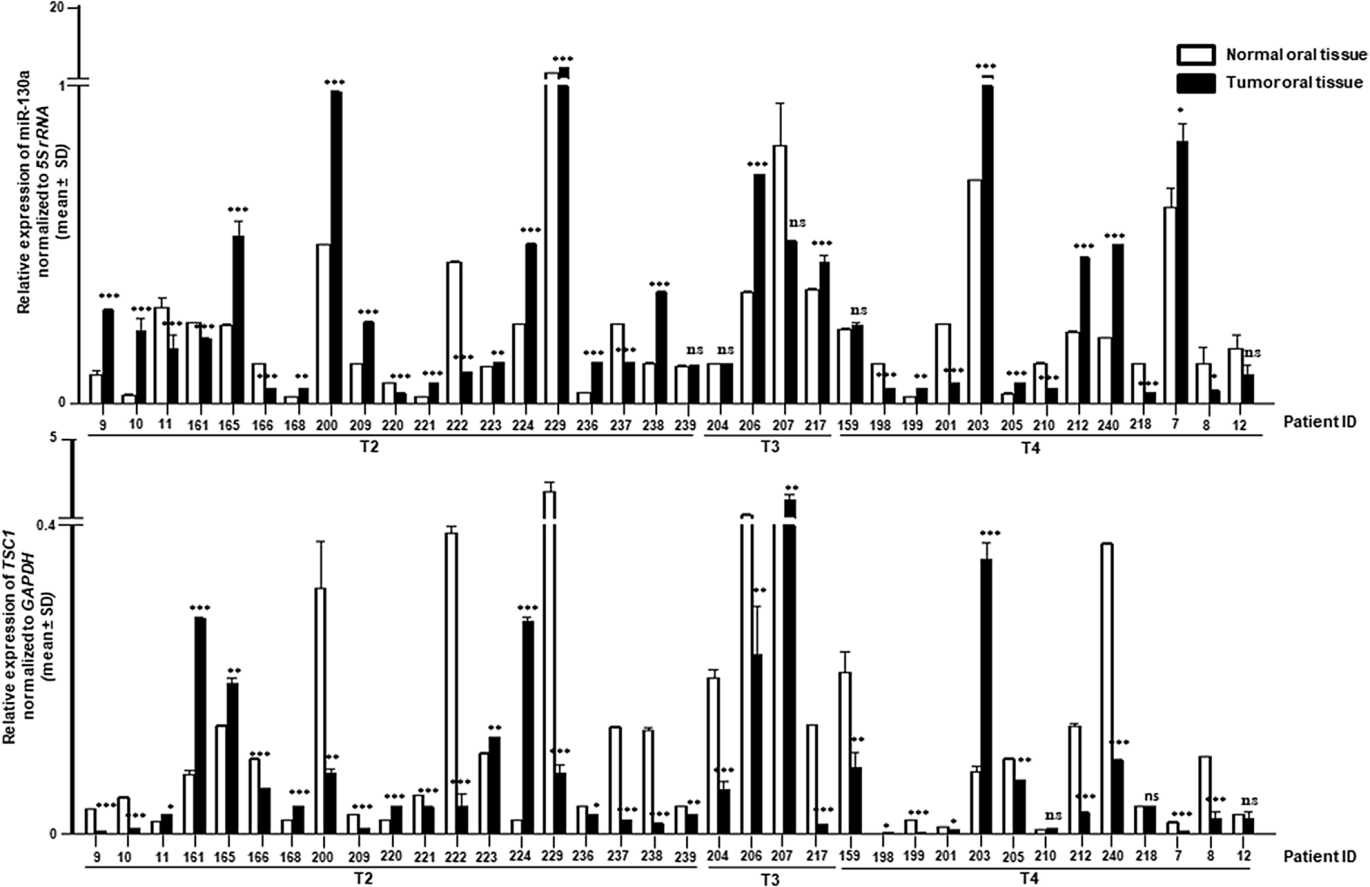
Correlation of miR-130a and TSC1 expression in OSCC patient samples. The graph represents the relative expression of miR-130a (upper panel) and TSC1 (lower panel) in 36 OSCC samples compared to their normal counterparts. Numbers along X-axis represent patients. T2, T3 and T4 represent stages of OSCC. Each bar is an average of two technical replicates. *, p < 0.05; **, p < 0.01; ***, p < 0.001; and ns, non-significant.

After validating *TSC1* as a target of miR-130a by bioinformatics analysis and in vitro assay, we analyzed the expression of miR-130a and *TSC1* by RT-qPCR in 36 OSCC samples. The results showed a significant upregulation of miR-130a in 20/36 OSCC samples and downregulation of *TSC1* in 23/36 OSCC samples as compared to their matched normal oral tissues (Fig. 4). However, miR-130a was downregulated in 11/36, and *TSC1* was upregulated in 10/36 samples. Overall, an inverse correlation was observed in the expression levels of miR-130a and *TSC1* in a majority of 19/36 (52.78%) OSCC samples (Fig. 4). These results also suggest that miR-130a-mediated post-transcriptional targeting of *TSC1* is a novel mechanism for its downregulation in OSCC.

### Function of TSC1 is dependent on the presence or absence of its 3′UTR

**Figure 5:**
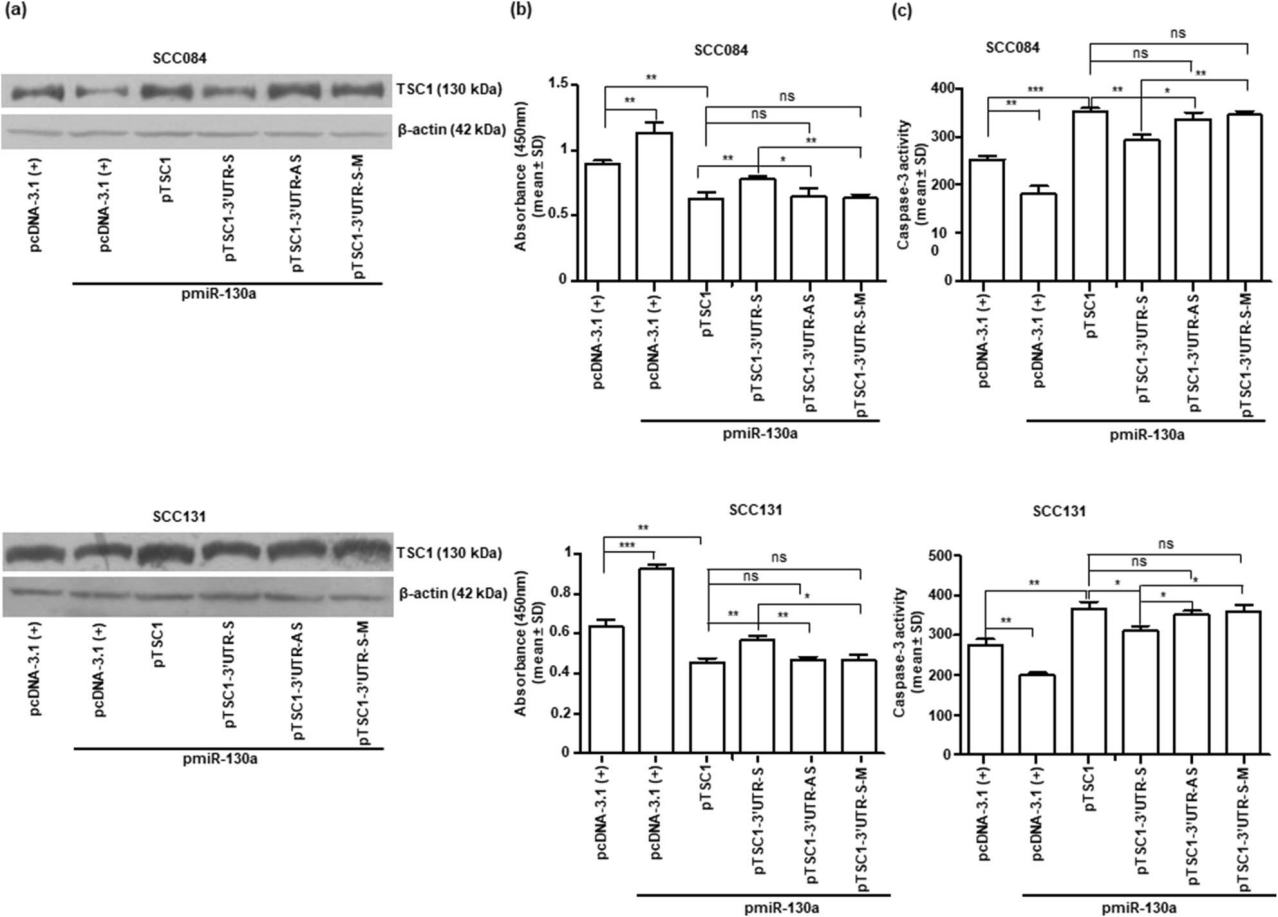
Function of TSC1 is dependent on the presence or absence of its 3′UTR in OSCC cells. (**a**) The Western blot analysis of OSCC cells co-transfected with pmiR-130a and different *TSC1* constructs (n = 2). Note, a reduced level of TSC1 in SCC084 and SCC131 cells co-transfected with pTSC1*-*3′UTR-S and pmiR-130a in comparison to those co-transfected with pmiR-130a and pTSC1*-*3′UTR-AS, pTSC1*-*3′UTR-S-M or pTSC1*,* underscoring that miR-130a targets *TSC1* by binding to its 3′UTR (full-length blots are presented in Supplementary Figure S7). (**b**) Cell proliferation by the BrdU cell proliferation assay. Note, a significantly increased rate of cell proliferation in SCC084 and SCC131 cells co-transfected with pTSC1*-*3′UTR-S and pmiR-130a as compared to those co-transfected with pTSC1*,* pTSC1*-*3′UTR-AS or pTSC1-3′UTR-S-M and pmiR-130a. Each bar is an average of 3 biological replicates. (**c**) Assessment of apoptosis by the caspase-3 activity assay. Note, a significantly decreased apoptosis in SCC084 and SCC131 cells co-transfected with pmiR-130a and pTSC1-3′UTR-S in comparison with those co-transfected with pmiR-130a and other *TSC1* constructs with 3′UTR. Each bar is an average of 3 biological replicates. *, *p˂*0.05; **, *p˂*0.01; ***, *p˂*0.001 and ns*,* non-significant.

To evaluate the role of *TSC1* 3′UTR on its expression and function, we generated different *TSC1* constructs by inserting the 3′UTR downstream to *TSC1*-ORF (pTSC1). The different *TSC1* constructs are as follows: the wild-type 3′UTR in a sense orientation (pTSC1*-*3′UTR-S), mutated 3′UTR in a sense orientation (pTSC1*-*3′UTR-S-M; all TSs mutated) and wild-type 3′UTR in an antisense orientation (pTSC1-3′UTR-AS). Next, we co-transfected different *TSC1* constructs with pmiR-130a separately in SCC084 and SCC131 cells and assessed the level of TSC1 by Western blotting (Fig. 5a). As expected, the results showed a reduced level of TSC1 in cells co-transfected with pTSC1-3′UTR-S and pmiR-130a in comparison to those co-transfected with pTSC1 and pmiR-130a, due to the presence of miR-130a TSs in pTSC1-3′UTR-S. Further, the level of TSC1 was increased in cells transfected with pTSC1-3′UTR-AS or pTSC1-3′UTR-S-M and pmiR-130a in comparison to those co-transfected with pTSC1-3′UTR-S and pmiR-130a, because of the absence of miR-130a TSs in pTSC1-3′UTR-AS or pTSC1-3′UTR-S-M (Fig. 5a).

Similarly, OSCC cells were transfected with the same combination of constructs as described above, and the changes in cell proliferation and apoptosis were assessed. As expected, the cell proliferation was significantly increased in cells co-transfected with pTSC1*-*3′UTR-S and pmiR-130a as compared to those co-transfected with pTSC1*,* pTSC1*-*3′UTR-AS or pTSC1-3′UTR-S-M (Fig. 5b) and pmiR-130a, confirming that miR-130a regulates cell proliferation, in part, by directly targeting the 3′UTR of *TSC1*. Also, the caspase-3 activity assay showed that the apoptosis was significantly decreased in cells co-transfected with pTSC1*-*3′UTR-S and pmiR-130a as compared to those co-transfected with pTSC1, pTSC1-3′UTR-AS or pTSC1-3′UTR-S-M and pmiR-130a (Fig. 5c), suggesting that miR-130a regulates apoptosis, in part, by targeting the 3′UTR of *TSC1*.

**Figure 6:**
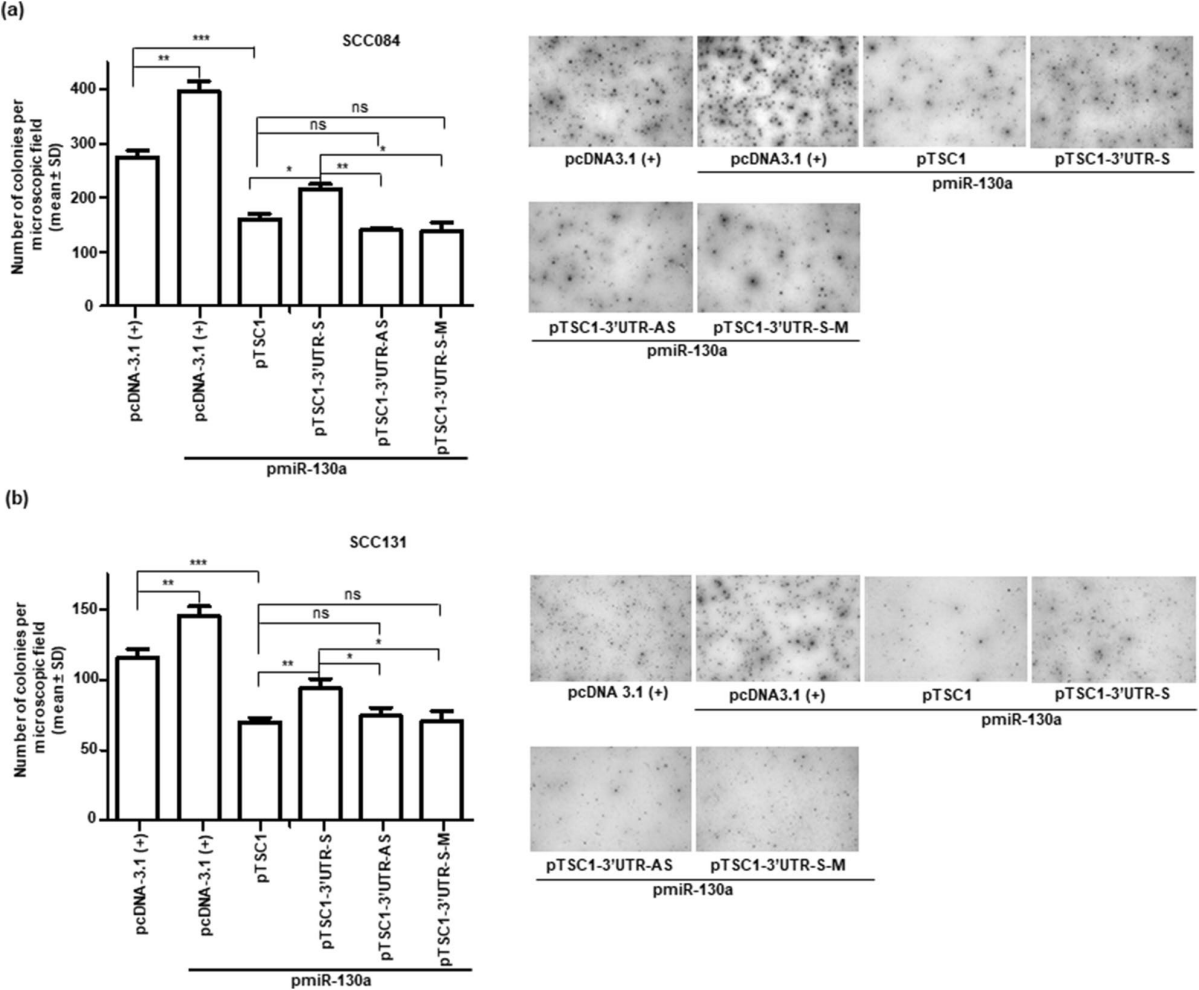
MiR-130a promotes soft agar colony formation by targeting TSC1 in OSCC cells. Assessment of the anchorage independent growth of SCC084 (**a**) and SCC131 cells (**b**) by the soft agar colony formation assay. Note, a significant increase in the number of colonies by cells co-transfected with pmiR-130a and pTSC1-3UTR-S in comparison with those co-transfected with pmiR-130a and pTSC1, pTSC1-3′UTR-AS or pTSC1-3′UTR-S-M constructs. The right panels represent the soft agar colony formation assay microphotographs. Each bar is an average of 3 biological replicates. *, p˂0.05; **, p˂0.01; ***, p˂0.001 and ns, non-significant.

**Figure 7:**
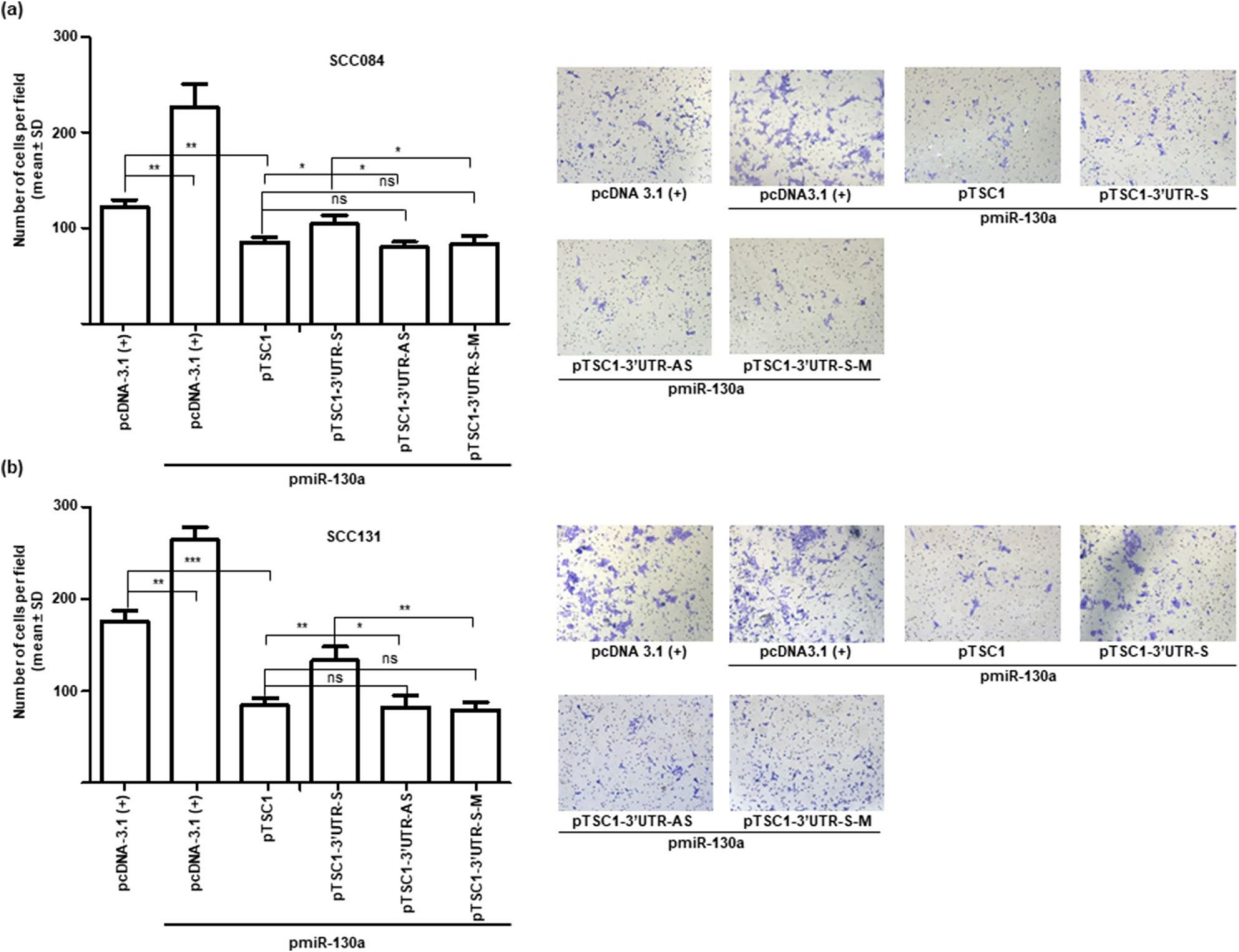
MiR-130a promotes cell invasion by via targeting TSC1 in OSCC cells. Analysis of the cell invasion of SCC084 (**a**) and SCC131 cells (**b**) by the matrigel invasion assay. Note, a significant increase in the invasion of cells co-transfected with pmiR-130a and pTSC1-3′UTR-S in comparison with those transfected with pmiR-130a and pTSC1, pTSC1-3′UTR-AS or pTSC1-3′UTR-S-M constructs. The right panels represent the matrigel invasion assay microphotographs. Each bar is an average of 3 biological replicates. *, p ˂ 0.05; **, p ˂ 0.01; ***, p ˂ 0.001 and ns, non-significant.

We also analysed the effect of miR-130a-mediated regulation of *TSC1* on anchorage independent growth and invasion of OSCC cells. We co-transfected pmiR-130a and different *TSC1* constructs (pTSC1, pTSC1*-*3′UTR-S, pTSC1-3′UTR-S-M and pTSC1-3′UTR-AS) or the vector separately in OSCC cells and observed the colony formation by the soft agar colony formation assay and invasion potential by the matrigel invasion assay. The results showed a significant increase in the number of colonies formed in soft agar (Fig. 6) and the number of invaded cells in the matrigel invasion assay (Fig. 7) for cells co-transfected with pmiR-130a and pTSC1-3′UTR-S as compared with those co-transfected with pmiR-130a and pTSC1*,* pTSC1*-*3′UTR-AS or pTSC1-3′UTR-S-M. These results suggested that miR-130a regulates anchorage independent growth and invasion of OSCC cells, in part, by targeting the 3′UTR of *TSC1.*

### Inhibition of miR-130a rescues the expression of TSC1

As miR-130a reduces the level of TSC1, we first optimized the dosage of antagomiR-130a sufficient to increase the level of TSC1 in SCC084 cells. The results showed that both 800 nM and 1200 nM dosages of antagomiR-130a were found to be effective in increasing the level of TSC1 (Supplementary Fig. S2). We then inhibited the endogenous level of miR-130a by antagomiR-130a (1200 nM) and analyzed its effect on mTORC1 activation. The Western blot analysis showed an increased level of TSC1 and a low level of p-S6K in antagomiR-130a treated SCC131 and SCC084 cells as compared to those treated with mock (Supplementary Fig. S3). However, there was no change in p-TSC2, TSC2 and S6K levels (Supplementary Fig. S3). The RT-qPCR analysis showed decreased levels of miR-130a in antagomiR-130a treated cells, confirming its specificity.

### Restoration of TSC1 expression by miR-130a inhibition suppresses tumor growth in vivo

**Figure 8:**
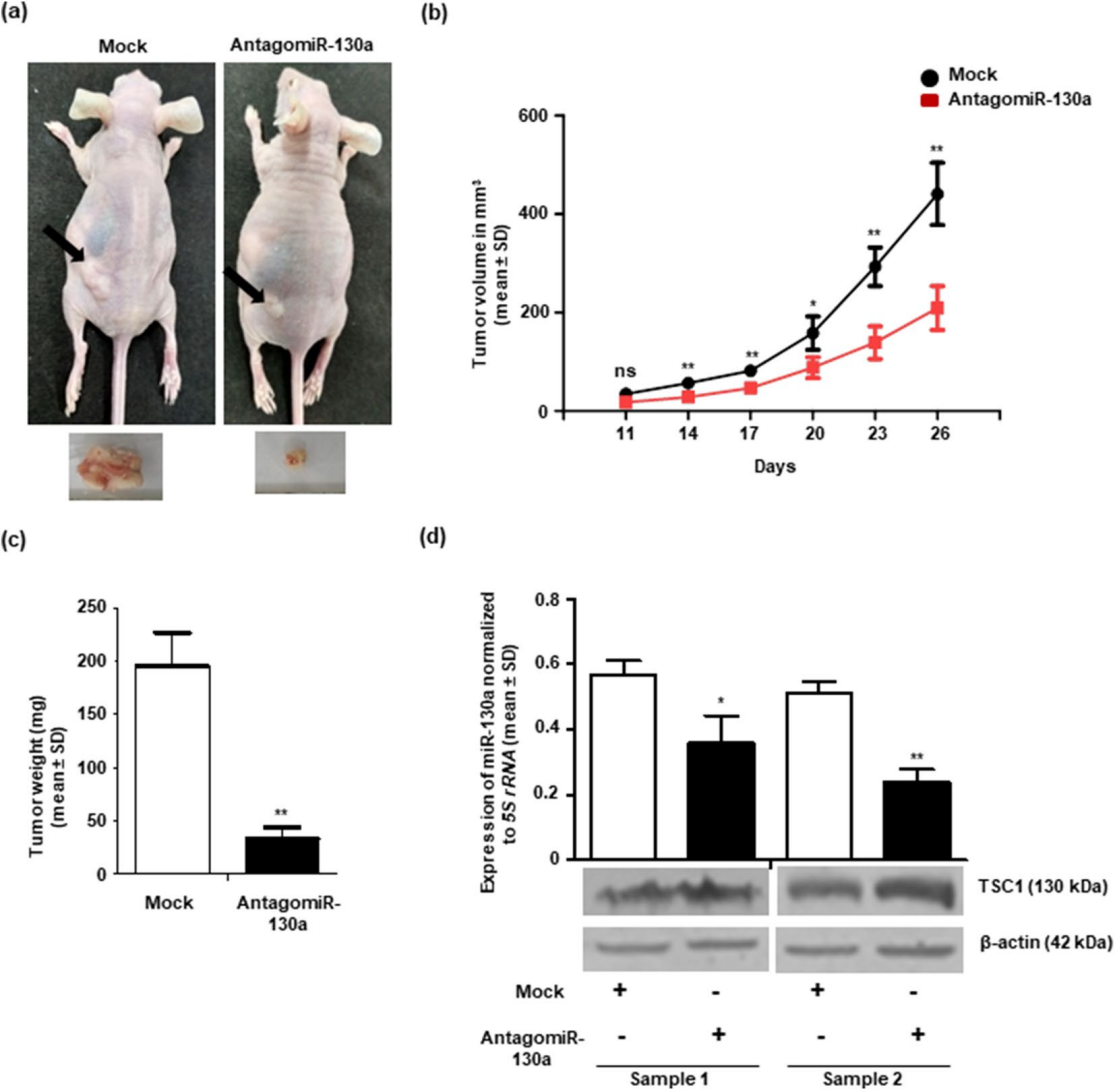
Inhibition of miR-130a level suppresses tumorigenicity in vivo. The effect of antagomiR-130a on SCC131 cell-derived xenografts in nude mice. (**a**) Top panel: photographs of nude mice show tumor growth after 26 days of injection. Bottom panel: excised xenografts on day 26. (**b**) Effect of miR-130a inhibitor on the volume of xenografts during a time course of 26 days. Each data point is an average of 3 xenografts. (**c**) Effect of miR-130a inhibitor on the weight of xenografts on day 26. Each bar is an average of 3 xenografts. (**d**) Western blot and RT-qPCR analysis to assess the level of TSC1 and miR-130a in xenografts on day 26 (n = 2). Sample 1 & 2 are different nude mice xenografts. Expression of miR-130a is an average of 2 technical replicates. *, p ˂ 0.05; **, p ˂ 0.01; and ns, non-significant (full-length blots are presented in Supplementary Figure S8).

As our in vitro studies showed the oncogenic role of miR-130a, we hypothesized that the depletion of miR-130a expression and thereby the restoration of tumor suppressor TSC1 levels in OSCC cells might have an anti-tumor effect in vivo. To this end, we used an in vivo pre-treated xenograft nude mouse model. Briefly, we transfected the antagomiR-130a (1200 nM) and scrambled oligos/mock (1200 nM) separately in SCC131 cells and injected them in left flanks of nude mice after 24 h of transfection and observed for tumor growth till 26 days. As expected, there was a significant reduction in both tumor volume and weight in nude mice injected with antagomiR-130a treated cells compared to that of mock control (Fig. 8a-c). Next, we assessed the levels of miR-130a and TSC1 in these xenografts by RT-qPCR and Western blot respectively. As expected, there was a decrease in miR-130a expression and an increase in TSC1 levels in xenografts treated with antagomiR-130a compared to mock treated ones (Fig. 8d), which further confirms that the reduced tumor growth in nude mice is due to the targeting of 3′UTR of *TSC1* by miR-130a.

### MiR-130a inhibits autophagy in OSCC cells through TSC1/mTOR axis

As TSC1 is known to regulate autophagy through mTORC1, we overexpressed miR-130a and TSC1 in OSCC cells and analyzed its effect on autophagy. The Western blot analysis showed that miR-130a suppressed autophagy as indicated by increased levels of p-ULK1 and p62, whereas pTSC1 transfected cells showed enhanced autophagy with decreased levels of p-ULK1 and p62 compared to vector transfected cells (Supplementary Fig. S4).

## Discussion

The current study was aimed to elucidate the role of miRNAs in the regulation of the tumor suppressor gene *TSC1* in OSCC. Previous reports have identified a few miRNAs (miR-451, miR-19a, miR-19b, miR-92b-3p and miR-126-3p) which directly target *TSC1* and thereby regulate its diverse cellular functions such as cell proliferation, apoptosis, migration and invasion^28–32^. In this study, we showed that the overexpression of miR-130a reduces the expression of *TSC1* at both transcript and protein levels in OSCC cells (Fig. 1b). Previously, Wang et al*.* have shown that the overexpression of miR-130a in ovarian cancer cells significantly reduced the expression of *TSC1* at the protein level^33^. By abrogating the three TSs in *TSC1* 3′UTR sequentially and in combinations, we were able to show for the first time that all of them contribute to sequence-specific interaction between miR-130a and *TSC1* 3′UTR (Fig. 2b).

MiR-130a, located on chromosome 11q12.1, plays an oncogenic role in several human cancers such as ovarian cancer, gastric cancer, salivary adenoid cystic carcinoma (SACC) and esophageal cancer and is strongly associated with lymph node metastasis and poor prognosis^33–38^. However, the association of miR-130a in the pathogenesis of OSCC and its underlying mechanism remain elusive. Here, we showed for the first time that miR-130a is upregulated in a majority of OSCC tumors as compared with their normal counterparts (Fig. 4). A study from our lab has already shown that *TSC1* is downregulated in OSCC^2^. The present study showed that *TSC1* is downregulated in a majority of OSCC samples as compared with their normal counterparts (Fig. 4)*.* Taken together, we observed an inverse correlation between the expression levels of miR-130a and *TSC1* in a majority of OSCC samples, suggesting the physiological significance of their interaction (Fig. 4).

Recent studies have focused on the emerging role of miRNAs in OSCC pathogenesis via aberrant activation of the PI3K/AKT/mTOR pathway^39,40^. Our study showed that overexpression of miR-130a increased p-S6K levels due to downregulation of TSC1, whereas the overexpression of pTSC1 led to a low level of p-S6K in both SCC084 and SCC131 cells (Fig. 3). We also assessed the levels of TSC2, as it is known that TSC1 stabilizes TSC2 and prevents it from degradation^41^. However, there was no change in TSC2 levels upon overexpression of miR-130a or pTSC1 in both SCC084 and SCC131 cells (Fig. 3), suggesting that TSC1 does not affect the expression of TSC2. This observation is in line with a previous study by Pradhan et al. where knockdown of *TSC1* by siRNA did not affect the TSC2 levels^42^. Also, the treatment of antagomiR-130a restored the expression levels of TSC1 and showed decreased mTOR activity in both SCC084 and SCC131 cells (Supplementary Fig. S3). All these observations clearly indicate that miR-130a deregulates the PI3K/AKT/mTOR pathway by targeting *TSC1*. Studies also suggest that TSC1 plays a prominent role in the regulation of autophagy^10^. Li et al. showed that miR-193b-3p, which also targets *TSC1*, regulates autophagy of NSC-34 neuronal cells through TSC1/mTOR axis^11^. Our results also show that miR-130a/TSC1/mTOR axis regulates autophagy in OSCC cells (Supplementary Fig. S4).

Previous reports show that changes in TSC1 or miR-130a expression is associated with the modulation of one or more of the cellular properties like proliferation, apoptosis, colony forming ability and invasion in several cancers such as prostate, multiple myeloma, gastric, SACC, and ovarian cancer^20,30,33–35,43,44^. Interestingly, as it is known that a single miRNA can target many genes and a gene can also be targeted by many miRNAs^45^, our next aim was to confirm whether the change in *TSC1* expression brought about by miR-130a is indeed an outcome of the interaction between the 3′UTR of *TSC1* and miR-130a. To achieve this goal, we have hooked up the sense, anti-sense or mutant 3′UTR of *TSC1* downstream to the *TSC1*-ORF and transfected these constructs (pTSC1*-*3′UTR-S, pTSC1-3′UTR-AS, pTSC1-3′UTR-S-M) separately in OSCC cells with pmiR-130a. This approach thereby facilitated to concomitantly confirm that the change in expression of TSC1 is due to the interaction between its 3′UTR and miR-130a, and aid in analyzing the downstream cascade of cellular events that were regulated by miR-130a via targeting of *TSC1.*

As expected, we observed a significant increase in cell proliferation (Fig. 5b), anchorage independent growth (Fig. 6) and invasion (Fig. 7) and a significant decrease in apoptosis (Fig. 5c) in OSCC cells co-transfected with pTSC1*-*3′UTR-S and pmiR-130a as compared to those co-transfected with pTSC1*,* pTSC1*-*3′UTR-AS or pTSC1-3′UTR-S-M and pmiR-130a, confirming that miR-130a regulates various cellular characteristics, in part, by directly targeting the 3′UTR of *TSC1*.

Wang et al. found an increase in the average tumor size and the number and volume of lung metastatic nodules in mice injected with A2780 or SKOV3 cells overexpressing miR-130a compared with control^33^. Yang et al*.* showed that tumor xenografts of the miR-130a inhibitor group were significantly smaller than those in the control group^46^. In our study, the mice injected with antagomiR-130a treated OSCC cells showed reduced tumor weight and volume compared to mock treated cells, which reveals the oncogenic function of miR-130a (Fig. 8). Taken together, these results suggested that miR-130a exerts its oncogenic functions in OSCC at least, in part, by regulating *TSC1.*

In summary, the present observations clearly demonstrate the oncogenic role of miR-130a in OSCC. We show that miR-130a represses the expression of TSC1 and thereby activates the PI3K/AKT/mTOR signaling. The RT-qPCR analysis showed that there was an inverse correlation in expression levels of miR-130a and *TSC1* in a majority of OSCC samples. Further, miR-130a increases cell proliferation, invasion and anchorage independent growth of OSCC cells by targeting the 3′UTR of *TSC1.* Our in vivo study suggests that the inhibition of the miR-130a via a synthetic antagomiR-130a may open new therapeutic options in the treatment of OSCC.

## Materials and methods

### In silico analysis of miRNAs targeting *TSC1*

Five miRNA target prediction programs (DIANA-microTv3.0, microRNA, miRDB, TargetScan and PicTar) were used to identify miRNAs targeting the 3′UTR of *TSC1* (NCBI Gene ID 7248) (Supplementary Table S1).

### Tissue samples

A total of 36 paired normal oral tissue and OSCC samples were collected from the HCG-Bangalore Institute of Oncology, Bangalore, India over the span of 10 yrs (2007 to 2017) (Supplementary Table S2). Tissue samples were stored in RNAlater (Sigma-Aldrich, St. Louis, MO) and frozen in a -80 °C freezer until use. Oral cancer patients enrolled in the study were not under any treatment at the time of the surgery. All the samples were obtained with informed consent from the patients and approval from the human ethics committee (# 01–09-2014) of the Indian Institute of Science, Bangalore. This study was conducted in accordance with principles of Helsinki declaration. OSCC samples were classified according to the TNM (Tumor, Node and Metastasis) classification based on the UICC (Union for International Cancer Control, Switzerland; http://www.uicc.org/resources/how-use-tnm-classification). Details of patients are given in Supplementary Table S2.

### RNA extraction and cDNA preparation

Total RNA including miRNA was isolated using TRI-Reagent (Sigma-Aldrich, St. Louis, MO), and quantitated using NanoDrop 1000 Spectrophotometer (Thermo Fischer Scientific, Waltham, MA). First-strand cDNA was synthesized using 2 µg of total RNA and a Verso cDNA Synthesis Kit (Thermo Fischer Scientific, Waltham, MA).

### RT-qPCR analysis

The expression level of miR-130a was determined by RT-qPCR as suggested by Sharbati-Tehrani et al*.*^47^. Details of the primers are given in Supplementary Table S3. The RT-qPCR analysis was carried out using the DyNAmo ColorFlash SYBR Green qPCR Kit in a StepOnePlus Real-Time PCR System **(**Thermo Fischer Scientific, Waltham, MA). *GAPDH* and *5S rRNA* were used as normalizing controls^48,49^. The following equation Δ*Ct*_gene_ = *Ct*_gene_—*Ct*_normalizing control,_ was used to calculate the fold change. *Ct* represents cycle threshold value, and Δ*Ct* represents the gene expression normalized to *GAPDH* or *5S rRNA*. A two-tailed unpaired *t-*test was performed using the GraphPad PRISM5 software (GraphPad Software Inc., San Diego) to analyze the statistical significance of the difference in mRNA expression.

### Plasmid constructs

MiR-130a (pmiR-130a) and *TSC1-*ORF (pTSC1) constructs were generated in pcDNA3-EGFP and pcDNA3.1 ( +) vectors, respectively, using human genomic DNA or cDNA as templates as required and gene specific primers (Supplementary Table S4) by a standard laboratory method. A 1,384 bp long 3′UTR of *TSC1* was cloned at the 3′end of the luciferase ORF in the pMIR-REPORT miRNA Expression Reporter Vector System (Thermo Fischer Scientific, Waltham, MA) in both sense and antisense orientations using human genomic DNA as a template. Primers were designed (Supplementary Table S4) using specific DNA sequences retrieved from the UCSC Genome Browser (https://genome.ucsc.edu), and PCR was performed using a standard laboratory procedure to facilitate directional cloning. The site-directed mutagenesis was carried out to generate constructs with the 3′UTR of *TSC1* harboring mutations in the TS1 (target site 1), TS2 (target site 2) or TS3 (target site 3) or all the TSs, according to Rather et al.^50^. The pTSC1-3′UTR-S, pTSC1-3′UTR-AS or pTSC1-3′UTR-S-M constructs were generated by cloning the relevant 3′UTR sequences downstream to the *TSC1-*ORF at *EcoR* V and *Not* I sites in the pTSC1 construct. All the constructs were sequenced on an ABI PRISM A310-automated sequencer (Thermo Fisher Scientific, Waltham, MA) to confirm the directionality and error-free sequence of the inserts.

### Cell culture

Human oral squamous cell carcinoma cell lines, UPCI: SCC084 and UPCI: SCC131, were a gift from Prof. Susanne Gollin, University of Pittsburgh, Pittsburgh, PA. Cells were maintained in DMEM supplemented with 10% FBS and 1X Antibiotic Antimycotic solution (Sigma-Aldrich, St. Louis, MO) at 37 °C in 5% CO_2_^50^.

### Transfection and reporter assays

SCC131 or SCC084 cells were seeded at a density of 2 × 10^6^ cells/well in a 6-well plate and transiently transfected with an appropriate construct or a combination of constructs using the Lipofectamine 2000 Transfection Reagent (Thermo Fisher Scientific, Waltham, MA). After 48 h, cells were harvested for either total RNA isolation using TRI-Reagent or total protein lysate preparation using the CelLytic M Cell Lysis Reagent (Sigma-Aldrich, St. Louis, MO). For the dual-luciferase reporter assay, 5 × 10^4^ cells/well were transfected with different constructs as mentioned above. The assay was carried out after 48 h of transfection in SCC084 cells, using the Dual-Luciferase Reporter Assay System (Promega, Madison, WI)^50^. The pRL-TK control vector, coding for Renilla luciferase, was co-transfected for normalizing the transfection efficiency ^50^.

### Western hybridization

Protein lysates from cells and tissue samples were prepared using the CelLytic M Cell Lysis Reagent (Sigma-Aldrich, St. Louis, MO). The proteins were resolved on an SDS-PAGE and then transferred to a PVDF membrane (Pall Corp., Port Washington, NY). The signal was visualized using an appropriate antibody and the Immobilon Western Chemiluminescent HRP substrate (Milipore, Billerica, MA). The anti-β-actin antibody (Cat# A5441) purchased from Sigma-Aldrich (St. Louis, MO) was used as a loading control. Antibodies such as anti-TSC1 (Cat# 6935S), anti-TSC2 (Cat# 3612), anti-phospho-p70S6 Kinase (Thr389) (Cat# 9205), anti-p70S6 Kinase (49D7) (Cat# 2708), anti-phospho-ULK1 (Ser757) (Cat# 6888) and anti-SQSTM1/p62 (Cat# 5114) were purchased from Cell Signalling Technologies (Danvers, MA). The anti-mouse HRP-conjugated secondary antibody (Cat# HP06) and anti-rabbit HRP-conjugated secondary antibody (Cat# HP03) were purchased from Bangalore Genei (Bangalore, India). The anti-phospho-TSC2 (S939) (Cat# ab52962) was purchased from Abcam (Cambridge, MA).

### Cell proliferation assay

CHEMICON BrdU Cell Proliferation Assay Kit (Milipore Corporation, Billerica, MA) was used to determine cell proliferation^48^. To this end, 2,000 cells were seeded in 96-well plates and transiently transfected with different constructs. The BrdU label was added at 2, 4, or 6 days and incubated for 20 h in a humidified CO_2_ incubator. The remaining protocol was followed as per the manufacturer’s instructions, and absorbance was measured at 450 nm using Infinite 200 PRO Plate reader (Tecan Group Ltd, Mannedorf, Switzerland).

### Apoptosis assay

The CaspGLOW Fluorescein Active Caspase-3 Staining Kit (Biovision, Mountain View, CA) was used to quantify the apoptosis of cells transfected with the appropriate constructs^50^. After transfection, FITC-DEVD label was added to cells, and rest of the steps were followed as per the protocol. The fluorescence intensity was measured, using Infinite 200 PRO Plate reader (Tecan Group Ltd, Mannedorf, Switzerland).

### Soft agar colony forming assay

The ability of cells to grow independently of solid surface, also known as anchorage independent growth, was assessed by the number of colonies formed in soft agar ^48,50^. After transfecting cells with appropriate constructs, they were harvested and 5,000 cells were plated in 1 ml of 0.35% Difco Noble Agar (Difco, Mumbai, India) diluted with culture media in a 35 mm dish. After 21 days, colonies were counted and imaged using Leica Inverted Microscope DMi1 (Leica Microsystems, Wetzlar, Germany).

### Cell invasion assay

The Corning BioCoat Matrigel Invasion Chamber was used for analyzing the invasion of cells (Corning Inc., Corning, NY) after transfection with different constructs^50^. Next, 50,000 cells were added to each transwell coated with Matrigel membrane (upper chamber) and placed in a chamber of 24-well plate containing 0.75 ml of culture media supplemented with 10% fetal bovine serum. Cells were allowed to invade by incubating them for 36 h in a humidified CO_2_ incubator. The non-invading cells were removed from the inside of transwell inserts using a cotton swab, and the invading cells were fixed using methanol and stained with 0.01% crystal violet (Sigma-Aldrich, St. Louis, MO)^50^. Cells were then imaged using Leica Inverted Microscope DMi1 and the number of cells invaded was estimated by counting in three random microscopic fields under a 10X objective^48^.

### Nude mouse xenograft model

To analyze the effect of miR-130a-mediated targeting of *TSC1* on tumor growth, 2 × 10^6^ SCC131 cells were transfected with 1,200 nM of antagomiR-130a (miR-130a inhibitor) or 1200 nM of mock (scrambled oligos) separately. After 24 h of transfection, cells from both groups were suspended separately in 150 μl of incomplete DMEM and then injected into the left flank of a female BALB/c athymic 5-week-old nude mouse subcutaneously^50^. A total of 12 nude mice, 6 per each group were injected with either mock or antagomiR-130a treated cells. Tumor growth was monitored by measuring its volume using a digital caliper every 3 days until 26 days^50^. The following equation was used to measure the tumor volume (*V*): *V* = (*W*^2^ X L)/2, where *L* and *W* represent length and width, respectively^48,50^. Excised tumors were weighed at the end of 26 days. Data is an average of three nude mice, and the experiment was performed with approval from the animal ethics committee (#CAF/Ethics/325/2013) of the Indian Institute of Science, Bangalore. All mice were maintained on a 12:12 h light/dark cycle, in proper cages with sufficient food and water. Animal experiments were performed in accordance with the National Institutes of Health Guide for the Care and Use of Laboratory Animals and ARRIVE guidelines. miRIDIAN microRNA hsa-miR-130a Hairpin Inhibitor/AntagomiR-130a (Cat# IH-300598–05-0005) and miRIDIAN microRNA Hairpin Inhibitor Negative Control #1/Mock (Cat# IN-001005–01-05) were purchased from Dharmacon (Lafayette, CO). All experiments were carried out in accordance with relevant guidelines and regulations.

## Supplementary Information

Supplementary Information
