# Supplementary material for: Oncogenic role of MiR-130a in oral squamous cell carcinoma
Source: Sci Rep. 2021 Apr 8;11:7787. doi: 10.1038/s41598-021-87388-4 (PMC8032739; doi:10.1038/s41598-021-87388-4)
Supplement: Supplementary file 1 — Supplementary Information [file 41598_2021_87388_MOESM1_ESM.pdf]

Oncogenic Role of MiR-130a in Oral Squamous Cell Carcinoma

Karthik Mallela[1], Swamy Shivananda[2], Kodaganur S. Gopinath[2] and Arun Kumar[1*]

[1]Department of Molecular Reproduction, Development and Genetics, Indian Institute of Science, Bangalore, 560012, India.
[2]HCG-Bangalore Institute of Oncology, Bangalore, 560027, India.

***Correspondence:**

Prof. Arun Kumar, Ph.D.
Department of Molecular Reproduction, Development and Genetics
Indian Institute of Science
Bangalore 560012
India
Tel: 91-80-2293 2998
Email: arunk@iisc.ac.in

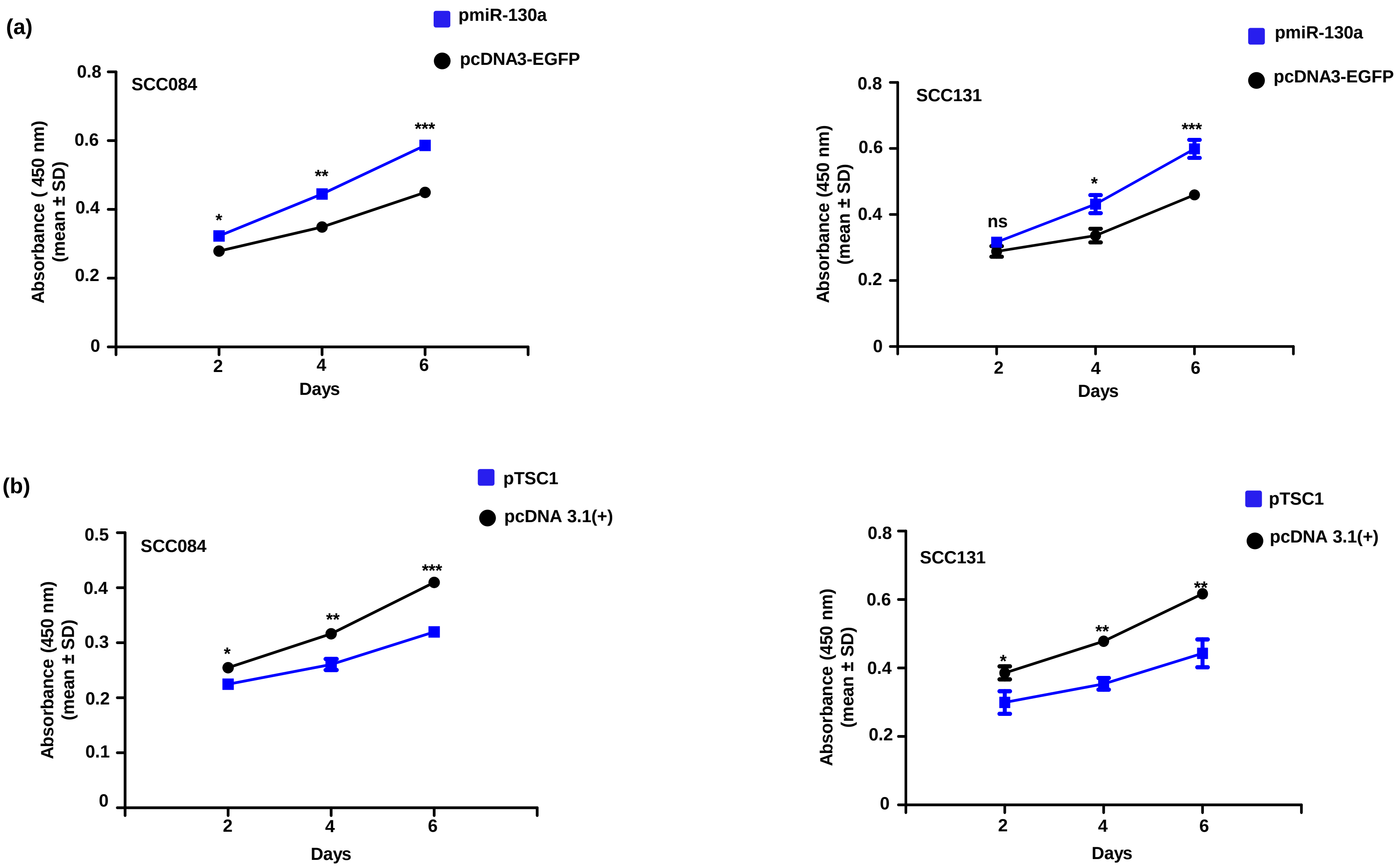

Supplementary Figure S1. Effect of miR-130a and TSC1 overexpression on cell proliferation using the BrdU cell proliferation assay. (a) An increase in cell proliferation was observed in miR-130a transfected SCC084 and SCC131 cells compared to the vector transfected cells (n=3). (b) A decrease in cell proliferation of SCC084 and SCC131 cells transfected with pTSC1 compared to the vector transfected cells (n=3). *, p<0.05; **, p<0.01; ***, p<0.001; and ns, non-significant.

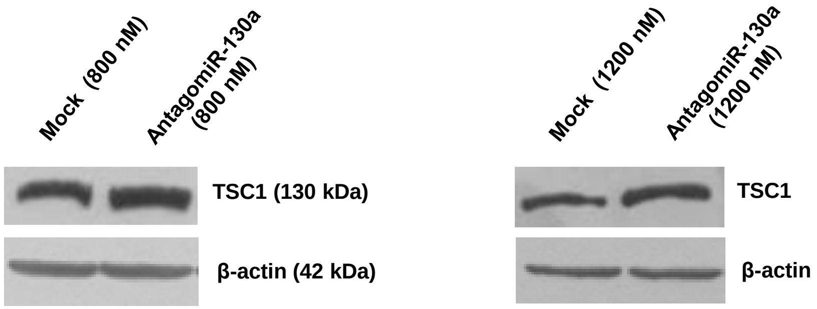

Supplementary Figure S2. Optimization of antagomiR-130a dosage in SCC084 cells. Cells were transfected with 800 nM and 1200 nM of antagomiR-130a and mock, followed by Western blot analysis using an anti-TSC1 antibody (n=2). Note, upregulation of TSC1 levels for both the dosages of antagomiR-130a (full-length blots are presented in Supplementary Figure S9).

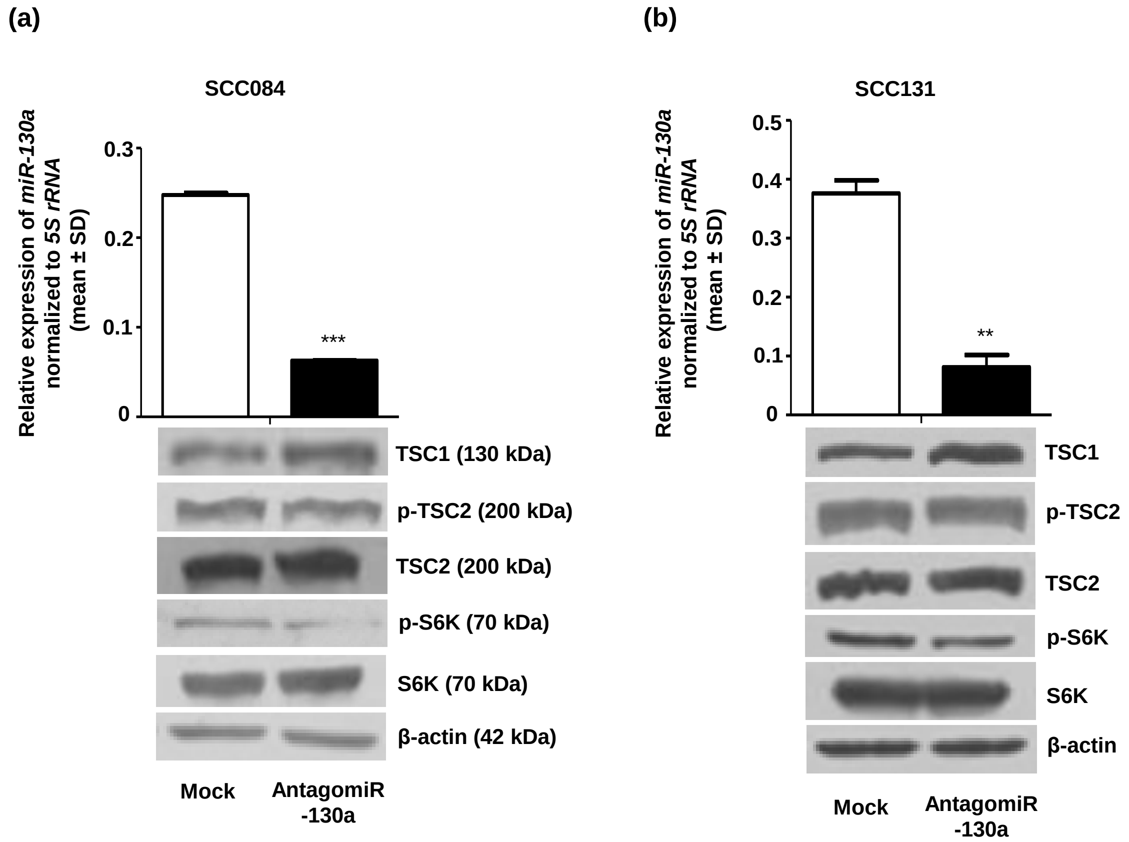

Supplementary Figure S3. Inhibition of miR-130a rescues the expression of TSC1. The Western blot analysis showed higher TSC1 and low p-S6K levels in antagomiR-130a (1200 nM) treated (a) SCC084 and (b) SCC131 cells as compared to mock (1200 nM) treated cells (n=2). However, there was no change in the levels of p-TSC2, TSC2 and S6K levels (n=2). The RT-qPCR analysis showed decreased levels of miR-130a in antagomiR-130a treated cells, confirming its specificity (n=2). β-actin and *5S rRNA* were used as normalizing controls (full-length blots are presented in Supplementary Figure S10).

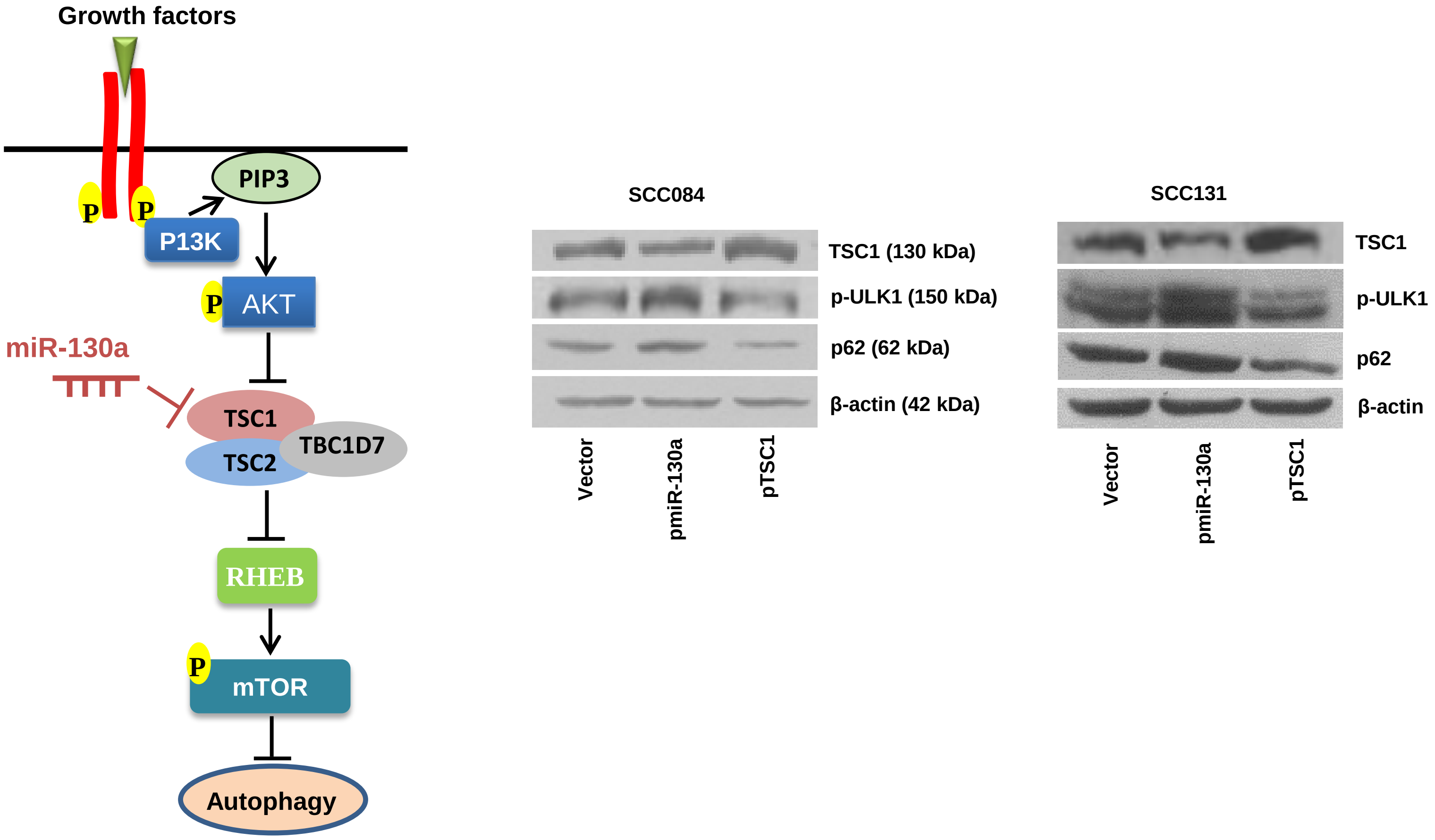

Supplementary Figure S4. MiR-130a inhibits autophagy in OSCC cells through TSC1/mTOR axis. The Western blot analysis showed higher p-ULK1 and p62 levels in pmiR-130a transfected cells compared to vector transfected cells, indicating that autophagy is suppressed (n=2). Whereas, pTSC1 transfected cells showed lower p-ULK1 and p62 levels compared to vector transfected cells, indicating enhanced autophagy (n=2) (full-length blots are presented in Supplementary Figure S11). The figure on the left was drawn using Microsoft PowerPoint version 2010. This figure is adapted from Jin *et al*.[51] & Jung *et al*.[52]

1a

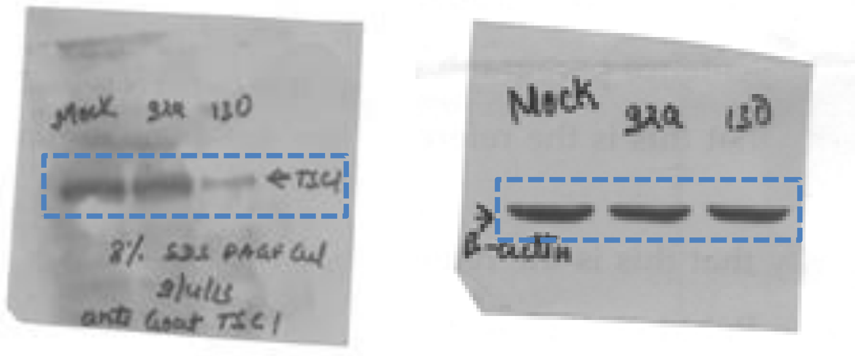

1b

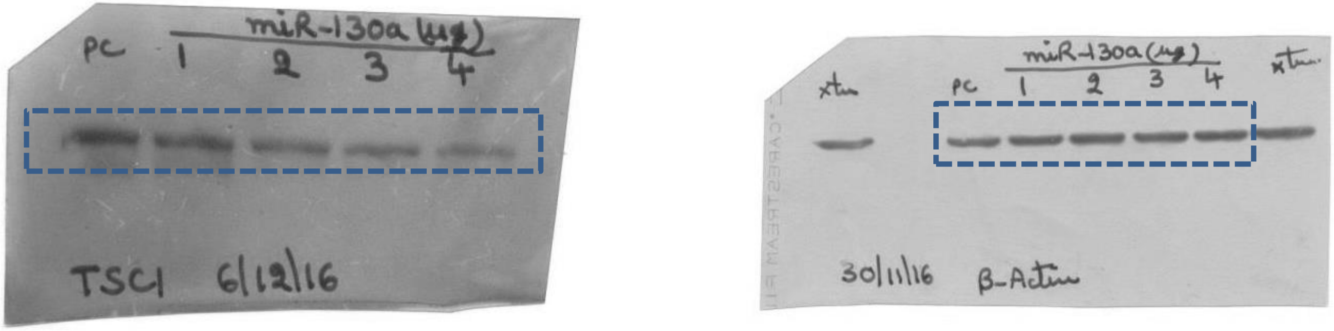

Supplementary Figure S5. Full-length blots of Figure 1.

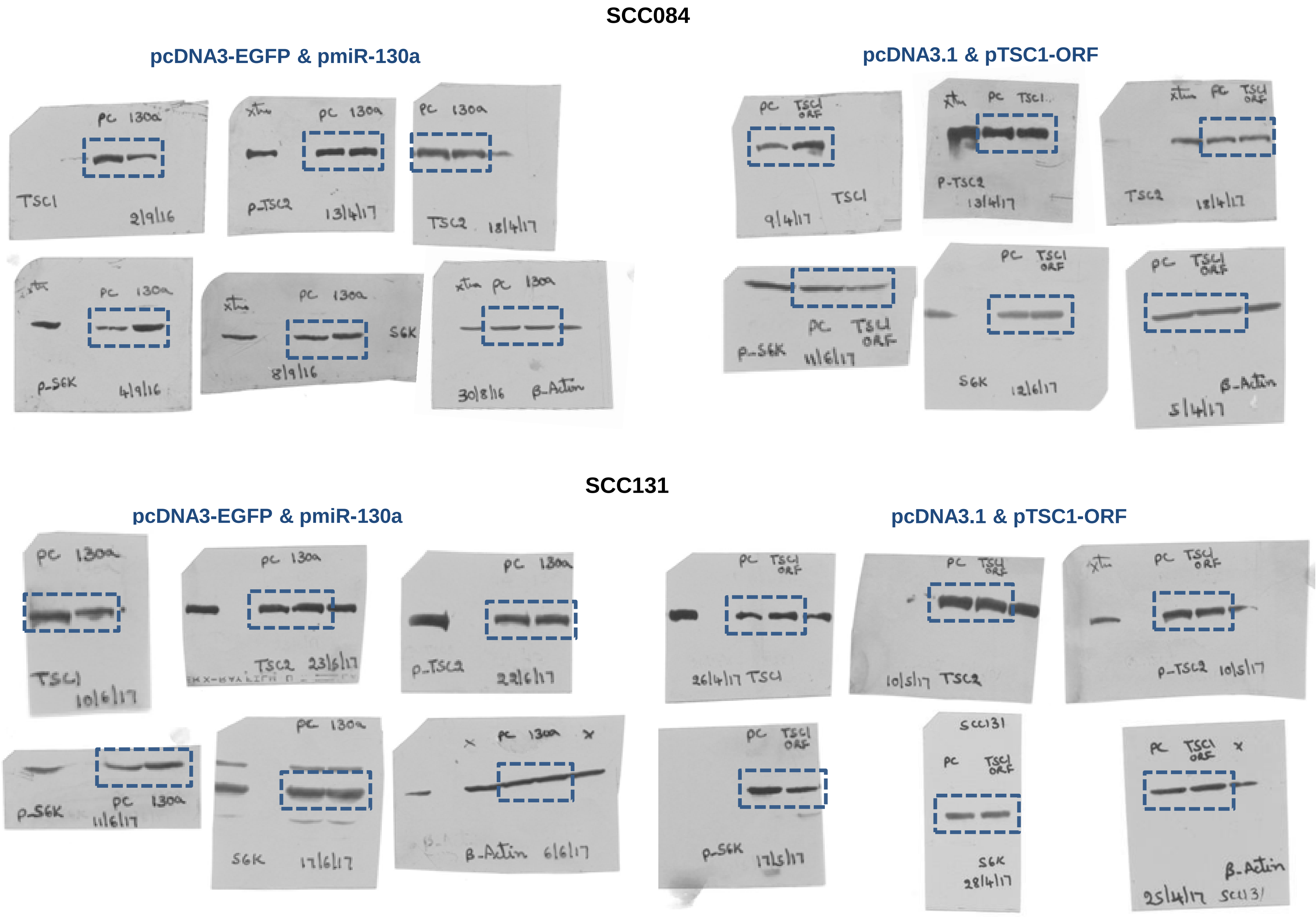

Supplementary Figure S6. Full-length blots of Figure 3.

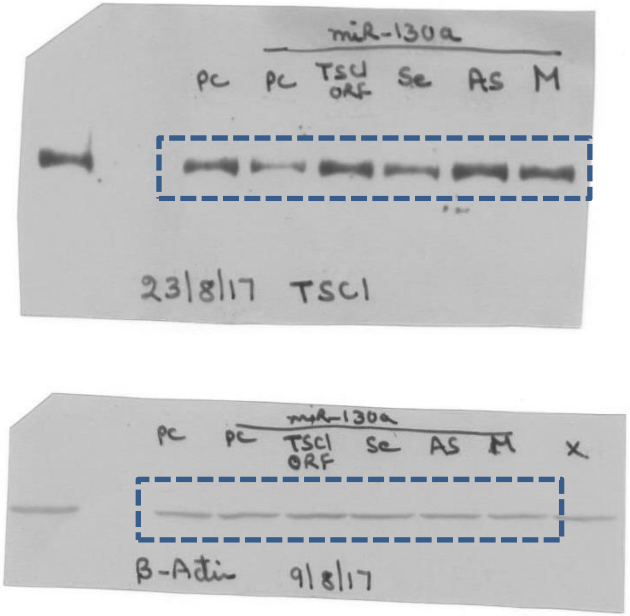

SCC084

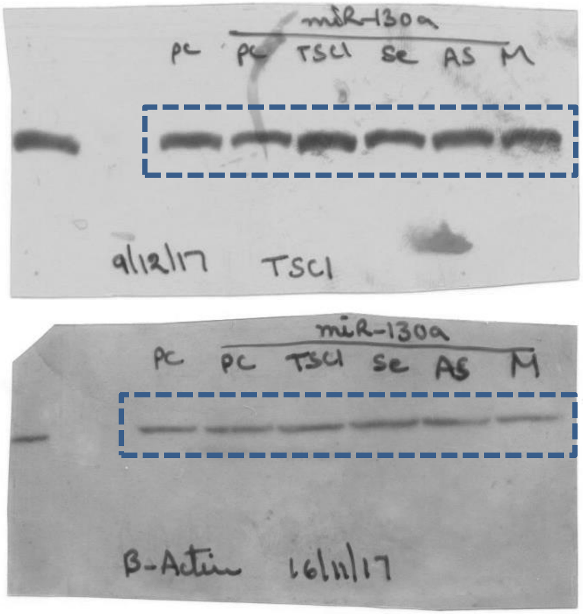

SCC131

Supplementary Figure S7. Full-length blots of Figure 5a.

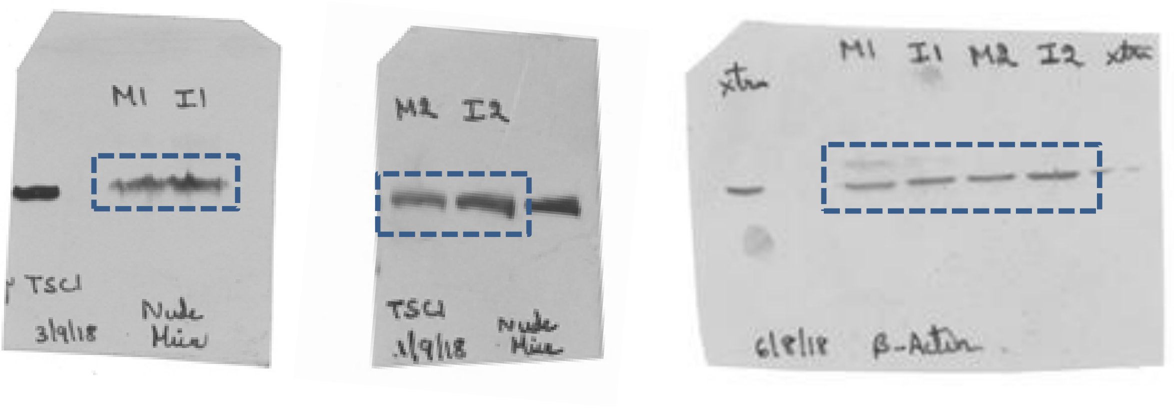

M: Mock (M1, M2)
I: Antagomir-130a (I1, I2)

Supplementary Figure S8. Full-length blots of Figure 8.

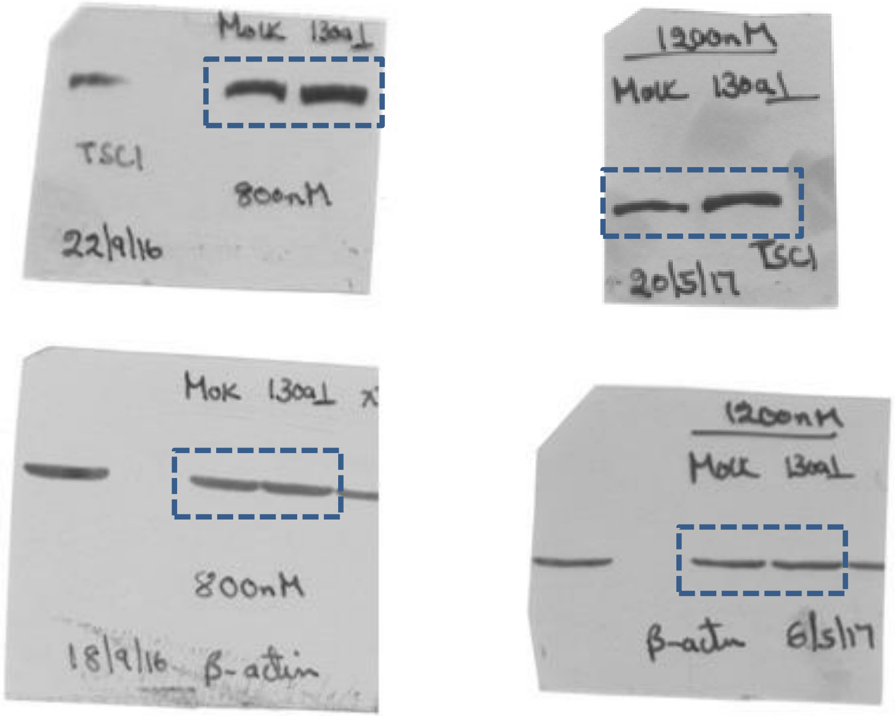

-ve control: Mock
130a⊥: Antagomir-130a

Supplementary Figure S9. Full-length blots of Supplementary Figure S2.

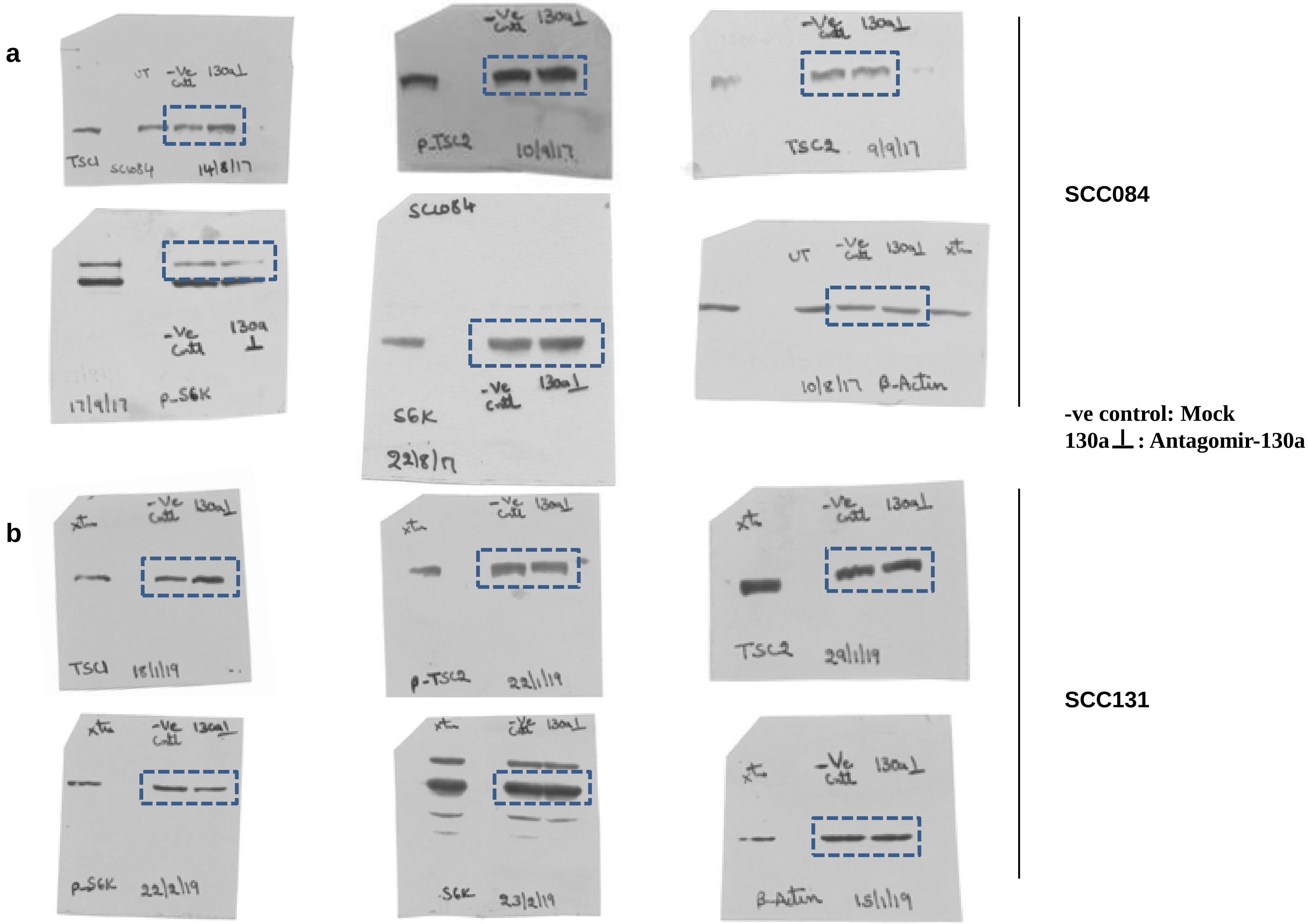

Supplementary Figure S10. Full-length blots of Supplementary Figure S3.

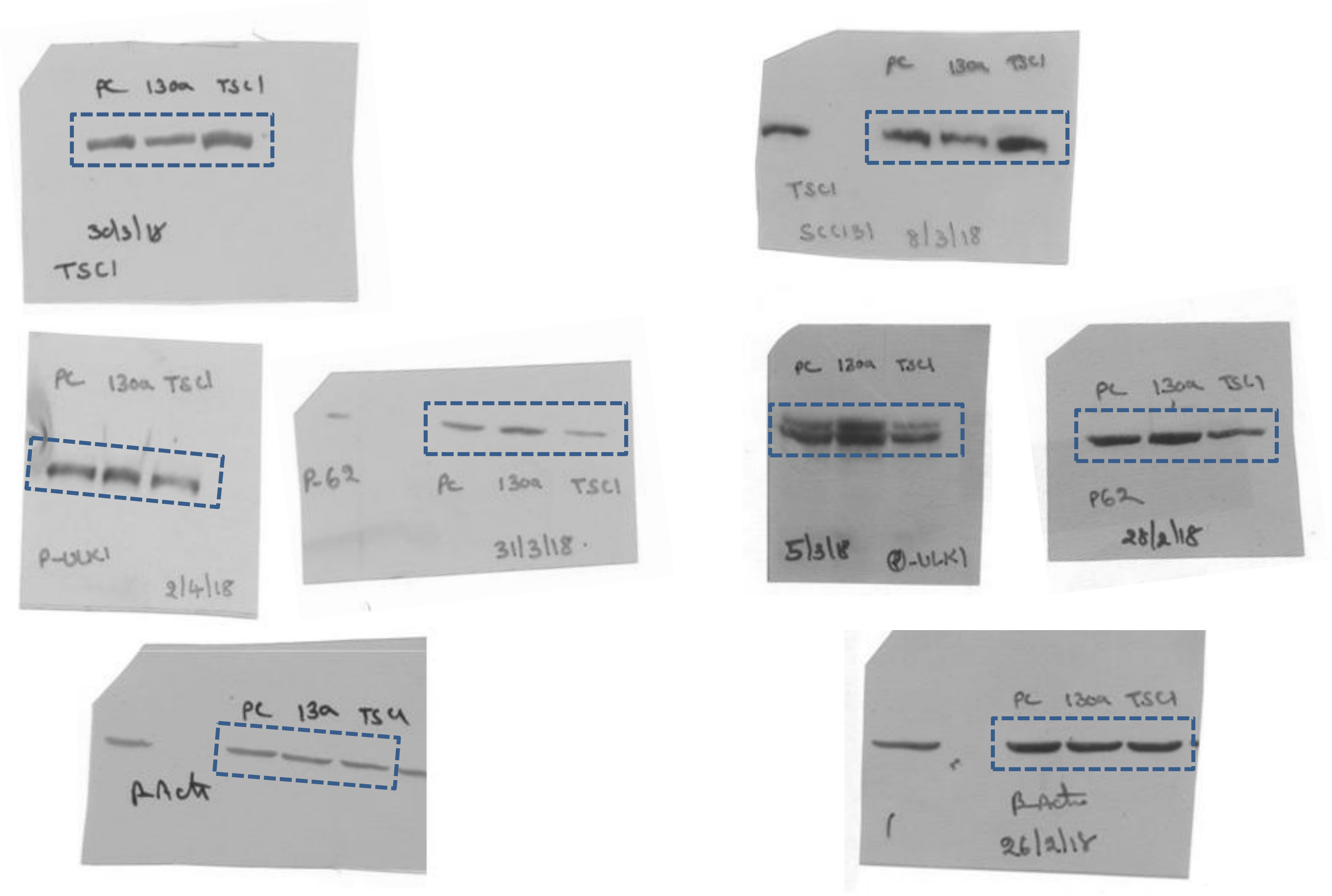

SCC084 **SCC131**

Supplementary Figure S11. Full-length blots of Supplementary Figure S4.

Supplementary Table S1. List of predicted miRNAs having potential binding sites in the 3'UTR of *TSC1*.

**miRNA prediction programs**				
**DIANA-microTv3.0**	**microRNA**	**miRDB**	**TargetScan**	**PicTar**
miR-15a	miR-19a	miR-16	miR-9a	miR-25
miR-16	miR-25	miR-17	miR-15a	miR-32
miR-19a	miR-27a	miR-19a	miR-18a	**miR-92a**
miR-27a	miR-32	miR-21	miR-19a	miR-98
miR-32	**miR-92a**	miR-27a	miR-25	miR-141
**miR-92a**	miR-98	miR-32	miR-30a	miR-200a
miR-98	miR-126	**miR-92a**	miR-32	miR-320
miR-103	**miR-130a**	**miR-130a**	**miR-92a**	miR-367
miR-107	miR-142	miR-141	miR-98	-
miR-128a	miR-181a	miR-149	miR-105	-
**miR-130a**	miR-183	miR-181a	miR-128	-
miR-301a	miR-202	miR-186	**miR-130a**	-
miR-320	miR-204	miR-193a	miR-183	-
miR-454	miR-211	miR-195	miR-193	-
miR-497	miR-301a	miR-204	miR-200b	-
-	miR-320a	miR-301a	miR-222	-

Supplementary Table S2. Clinicopathological parameters of the patients.

Sl. #	Pt. #	Age (yr)	Sex	Site of Cancer	TNM	Differentiation	Tobacco Usage
1	7	46	M	Right retromolar trigone	T4aN2bMx	NA	Nil
2	8	56	M	Right retromolar trigone	T4N0M0	NA	Nil
3	9	65	F	Right retromolar trigone	T2N0M0	NA	Nil
4	10	66	M	Left lateral border of tongue	T2N0M0	NA	Nil
5	11	64	M	Left retromolar trigone	T2N0Mx	NA	Nil
6	12	66	F	Carcinoma of oral cavity	T4N1M0	NA	Nil
7	159	40	F	Carcinoma of right low gingivobuccal sulcus	T4N0M0	NA	Nil
8	161	42	F	Carcinoma of tongue right	T2N1M0	Well	Tobacco chewing
9	165	50	F	Carcinoma of base of tongue	T2N1M0	Well	Smoker
10	166	50	F	Carcinoma of right buccal mucosa	T2N1M0	Well	Tobacco chewing
11	168	50	F	Carcinoma of right buccal mucosa	T2N1M0	NA	Tobacco
12	198	40	F	Carcinoma of buccal mucosa	T4aN2bM0	Well	Tobacco chewing
13	199	50	M	Carcinoma of buccal mucosa	T4aN2bM0	Well	Tobacco chewing
14	200	39	F	Carcinoma of left buccal mucosa	T2N1M0	Well	Betel chewing
15	201	55	F	Carcinoma of right buccal mucosa	T4aN1M0	NA	Nil
16	203	38	M	Carcinoma of buccal mucosa	T4aN1M0	Well	Tobacco & Alcohol
17	204	70	M	Carcinoma of right buccal mucosa	T3N1M0	Well	Tobacco chewing
18	205	60	F	Carcinoma of buccal mucosa	T4N1M0	Well	Tobacco chewing
19	206	44	M	Carcinoma of tongue right lateral border	T3N2bM0	Well	Tobacco chewing
20	207	60	F	Carcinoma of tongue	T3N1M0	Poor	Bedi smoker
21	209	72	M	Carcinoma of left lip	T2N0M0	NA	Tobacco
22	210	59	M	Left retromolar trigone	T4N2M0	NA	Tobacco
23	212	65	F	Carcinoma of right buccal mucosa	T4N1M0	Moderate	Tobacco
24	217	45	F	Carcinoma of left buccal mucosa	T3N1M0	Moderate	Betel chewing
25	218	56	F	Carcinoma of gingivobuccal sulcus and lower alveolus	T4N1M0	Well	Nil
26	220	37	M	Carcinoma of tongue right lateral border	T2N1M0	NA	Nil
27	221	53	M	Carcinoma of left buccal mucosa	T2N0M0	NA	Tobacco
28	222	50	M	Carcinoma of tongue right side	T2N1M0	Moderate	Nil
29	223	65	M	Carcinoma of right buccal mucosa	T2N1M0	Well	Tobacco
30	224	39	F	Carcinoma of tongue left side	T2N1M0	Well	Nil

31	229	51	M	Carcinoma of left buccal mucosa	T2N2M0	Moderate	Nil
32	236	32	M	Carcinoma of tongue right side	T2N1M0	Well	Nil
33	237	45	F	Carcinoma of right buccal mucosa	T2N3M0	Moderate	Tobacco
34	238	46	F	Carcinoma of right maxilla	T2N1M0	Moderate	Nil
35	239	52	M	Carcinoma of tongue	T2N0M0	Moderate	Nil
36	240	70	F	Carcinoma of right maxilla	T4N2M0	Poor	Nil

Abbreviations: TNM, Tumor Node Metastasis status; NA, not available.

Supplementary Table S3. Details of primers used in RT-qPCR.

Gene	Sequence (5' to 3')	Use of primers	Amplicon size (bp)	Annealing temp (°C)	Reference
TSC1	**F:** AAGCATAAGCTCTCTCAAGCACCTC<br>**R:** CCAAGACGCCTGTTGTGAGGACA	RT-qPCR	93	59	-
GAPDH	**F:** GAAGGGTGAAGGTCGGAGTC<br>**R:** GAAGATGGTGATGGGATTTC	RT-qPCR	226	60	-
(*MIR130A*) miR-130a	**RT6-miR-130a-3p:** TGTCAGGCAACCGTATTCACCGTGAGTGG TATGCCC<br>**Short miR-130a-3p-rev:** CGTCAGATGTCCGAGTAGAGGGGGAACG GCG CAGTGCAA TGTTAAAAGGG	RT-qPCR	-	-	[28]
	**MP-fw:** TGTCAGGCAACCGTATTCACC<br>**MP-rev:** CGTCAGATGTCCGAGTAGAGG	RT-qPCR	-	-	
5S rRNA	**F:** GCCCGATCTCGTCTGATCT<br>**R:** AGCCTACAGCACCCGGTATT	RT-qPCR	94	60	[28]

Abbreviations: F, forward primer; R, reverse primer; and bp, base pair; RT-qPCR, quantitative reverse transcription PCR.

Supplementary Table S4. Details of constructs generated in the present study.

Construct	Cloning vector	Primer sequence (5' to 3')	Amplicon size (bp)	Annealing temp. (°C)
pmiR-130a	pcDNA3-EGFP	**F:** CGAAAGCTTAAGAAGGCGGCAAAAGGAAGAG<br>*Hind* III<br>**R:** TGACTCGAGGCACTGCATACAGAAGTAGTTC<br>*Xho* I	397	60
p3'UTR-S	pmiR-REPORT	**F:** AGCTACGATATCAGACGCGTACCAAATAGTAGAGCATACCACTTG<br>*Mlu* I<br>**R:** TGTCGAGCGGCCGCATGTTTAAACGTAGTGCAAAACAATAAATTTTTATTTG T<br>*Pme* I	1384	58
p3'UTR-AS	pmiR-REPORT	**F:** TGTCGAGCGGCCGCATGTTTAAACACCAAATAGTAGAGCATACC ACTTG<br>*Pme* I<br>**R:** AGCTACGATATCAGACGCGTGTAGTGCAAAACAATAAATTTTTAT TTGT<br>*Mlu* I	1384	58
p3'UTR-S-M1	pmiR-REPORT	**F:** CCCAAGGCATTGATTTAGTTTGATTAAGCTTTTGTC<br>**R:** GACAAAAGCTTAATCAAACTAAATCAATGCCTTGGG	1384	56
p3'UTR-S-M2	pmiR-REPORT	**F:** GGCAGCCACGCATTTTTAGTTAGGAAGAATCAGTAATC<br>**R:** GATTACTGATTCTTCCTAACTAAAAATGCGTGGCTGCC	1384	56
p3'UTR-S-M3	pmiR-REPORT	**F:** ATAAAAATTTATTGTTTTAGTTACGTTTAAACATGCGGCCGC<br>**R:** GCGGCCGCATGTTTAAACGTAACTAAAACAATAAATTTTTAT	1384	60
p3'UTR-S-M1M2	pmiR-REPORT	Generated using primers as reported for p3'UTR-S-M1 and p3'UTR-S-M2 constructs	-	-
p3'UTR-S-M1M3	pmiR-REPORT	Generated using primers as reported for p3'UTR-S-M1 and p3'UTR-S-M3 constructs	-	-
p3'UTR-S-M2M3	pmiR-REPORT	Generated using primers as reported for p3'UTR-S-M2 and p3'UTR-S-M3 constructs	-	-
p3'UTR-S-M1M2M3	pmiR-REPORT	Generated using primers as reported for p3'UTR-S-M1, p3'UTR-S-M2 and p3'UTR-S-M3 constructs	-	-
pTSC1	pcDNA3.1 (+)	**F:** TGACGGTACCATGGCCCAACAAGCAAATGTCGGG<br>*Kpn* I<br>**R:** TGACGATATCTTAGCTGTGTTCATGATGAGTCTCA<br>*EcoR* V	3495	56
pTSC1-3'UTR-S	pcDNA3.1 (+)	**F:** AGCTACGATATCAGACGCGTACCAAATAGTAGAGCATACCACTTG<br>*EcoR* V<br>**R:** TGTCGAGCGGCCGCATGTTTAAACGTAGTGCAAAACAATAAATT TTTATTTGT<br>*Not* I	1384	58
pTSC1-3'UTR-AS	pcDNA3.1 (+)	**F:** TGTCGAGCGGCCGCATGTTTAAACACCAAATAGTAGAGCATACCACTTG<br>*Not* I<br>**R:** AGCTACGATATCAGACGCGTGTAGTGCAAAACAATAAATTTTTAT TTGT<br>*EcoR* V	1384	58
pTSC1-3'UTR-S-M	pcDNA3.1 (+)	Generated by ligating insert from p3'UTR-S-M1M2M3 to downstream of *TSC1*-ORF in pTSC1	-	-

Abbreviations: F, forward primer; R, reverse primer; and bp, base pair.
